# Direct PCR amplification from saliva sample using non-direct multiplex STR kits for forensic DNA typing

**DOI:** 10.1038/s41598-021-86633-0

**Published:** 2021-03-29

**Authors:** Pankaj Shrivastava, Toshi Jain, R. K. Kumawat

**Affiliations:** 1 DNA Fingerprinting Unit, State Forensic Science Laboratory, Sagar, MP 470001 India; 2 DNA Division, State Forensic Science Laboratory, Rajasthan, Jaipur 302016 India

**Keywords:** Biological techniques, Genetic techniques

## Abstract

Due to its proficiency to provide the most discriminating results for forensic applications, medical research and anthropological studies, multiplex PCR based STR analysis has been established as the most efficient technique in the forensic DNA analysis. Several multiplex amplification kits based on 4, 5 and 6 dyes chemistry are commercially available and used in forensic DNA typing across the globe. These multiplex PCR systems are routinely used for amplification of multiple STR loci (Autosomal, Y and/or X STR’s) in the DNA extracted from various biological samples. In the routine forensic DNA testing, DNA profile obtained is compared with the DNA profile of the reference sample, which takes a certain turnaround time and employs costly lab resources. Successive development in forensic DNA typing have resulted in advent of improved multiplex kits which have reduced the effective analysis time, cost and minimized the number of steps required in comparison to conventional forensic DNA typing. Specialized direct amplification compatible multiplex kits are also available nowadays. These kits are relatively costlier but still require few pre-processing steps, which does not make them worth the hefty cost. Herein, this study, we have used non-direct multiplex STR kits to assess their efficacy for direct amplification. In the present study, 103 saliva samples were directly amplified without any pre-treatment of the samples using thirteen non-direct multiplex kits (4 dyes, 5 dyes and 6 dyes chemistry based) for forensic DNA typing. Here, we report a validated direct PCR amplification protocol from the reference saliva samples by omitting DNA extraction and quantification steps, which resulted in 80% reduction of the turnaround time. The developed protocol is cost effective, time efficient and it does not compromise with the quality of DNA profiles. To the best of our knowledge, this is the first report for direct amplification of DNA with the most commonly used non-direct multiplex STR kits without any pre-treatment of the sample. Complete DNA profiles matching all the essential quality parameters were obtained successfully from all the tested samples.

## Introduction

**Figure 1:**
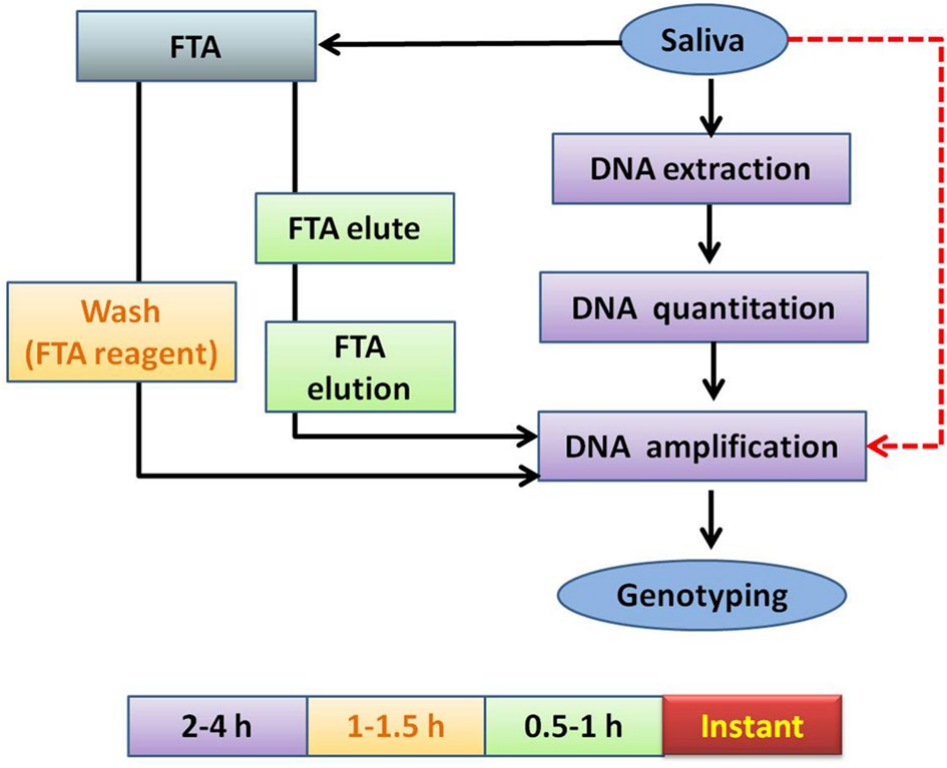
Workflow of conventional forensic DNA typing.

In early 1990s, the advent of the polymerase chain reaction (PCR) technology and the use of short tandem repeat (STR) polymorphism^1^ was a major breakthrough in forensic DNA technology. In last two decades, PCR-based STR typing has become a routine technique in the forensic investigations due to associated features such as specificity, sensitivity, feasibility to simultaneously amplify several loci (multiplexing) and automation^2,3^. Despite several improvements in the methodology, time required for the conventional forensic STR analysis could not be reduced and the process is still lengthy needing hours to several days for processing of most of the samples^4^. Conventional forensic DNA typing is a multistep process involving several steps of DNA extraction, quantification, amplification, genotyping and analysis (Fig. 1). These steps are not just time consuming but also result in loss of the valuable DNA^5,6^, which is already in minute quantities in the forensic samples. Also multiple processing steps need more human intervention which further increases the possibility of contamination and error. If these steps are reduced and the amplification could be achieved without the extraction, quantification, and concentration processes, higher yield of DNA could be achieved. Also, the chances of manual error and contamination could be reduced. Approximately 20–76% of DNA is lost from the swab samples during the DNA extraction step^7^. Various studies have reported that column-based DNA extraction techniques also result in the loss of DNA, thereby affecting the genotyping using this method^8–14^. Most of the time in routine forensic DNA typing is consumed in the pre-processing steps of DNA extraction and Quantitation^4^. The development of semi-automated DNA extraction kits and automated DNA extraction systems using pre-formulated kits has been a major breakthrough in this area so far. Some short non-automated extraction protocols like Chelex or alkaline lysis method and/or magnetic, paramagnetic beads based rapid DNA isolation are also in use. Since last decade, most of the forensic laboratories have encountered a considerable rise in the number of forensic DNA casework^15–17^. Towards the effort to avoid the delay in justice, a lot of research work has been conducted to improve the analysis speed and to develop faster processing methods to save the reporting time. Development of rapid, compact and portable devices capable of producing real-time results from forensic samples at the crime scene has been emphasised upon^15–17^. However, these efforts were not of much help to reduce the cost and time efficiency of the forensic DNA analysis, therefore were not adopted in most of the laboratories^18^. The analysis of mandatory reference samples in the forensic cases, which may vary from one to many, also require a similar lengthy processing time, as that of the case samples. This drawback is associated with both the methods viz. automated or specific direct amplification compatible chemistries^19^ which adds to the further delay in reporting the case. To curb down the analysis time and fasten the process of case reporting, there has been a growing interest towards direct amplification of case and reference samples such as blood and buccal swabs, single hair follicle and tape lifts of clothing, swab on FTA card^20–24^, blood^25^, fabrics^6^, hairs^26,27^, touch DNA^28,29^, blood stain^30^, fingernails^31^, tissues^32,33^ and fibers^34^. Direct amplification protocol eliminates the DNA extraction and quantitation steps, and accommodates the sample directly to the PCR step. The streamlined process of direct amplification for processing the DNA samples has obvious benefits such as simplify the process, low risk of contamination due to less handling steps, cost efficiency and has low chances of loss of forensic DNA, which already is in minute quantities^32,33^. Methods to successfully genotype different samples from varied origins in a single shot by direct amplification sounds promising and challenging too. Forensic cases have various sorts of circumstantial exhibits which could include anything found at the scene of crime. One such forensic case exhibits are buccal swabs and in the case, the subjects had a habit of smoking, chewing tobacco etc., it could cause inhibition in the process of PCR. Although, the buccal cells are stable for weeks or months if stored in the laboratory at – 20 °C till final processing, they are quite vulnerable to the bacterial growth, nucleic acid degradation. Reduced amplification ability after a span of 4 days or so has been observed, particularly under warm and moist conditions^35^. Keeping these points into consideration, saliva sample was chosen for the direct analysis. The fact that saliva or any cell suspension is expected to have some cell free DNA formed the ground for this study. Also, another important aspect considered was the nature and texture of the saliva cells as these cells are not strong enough to stand high temperatures like 95 °C, which is a prerequisite in most of the amplification kits for activation of hot start Taq polymerase^36^.

The process of genotyping nowadays does not require the high yield of DNA. This could be attributed to the improvement in the amplification step by using the upgraded and highly sensitive STR kits which require very low inputs of DNA (approx less than 500pg). The advantages associated with these advanced kits not only include the increase in the number of loci to be examined, as per the recommendation of the Scientific Working Group on DNA Analysis Methods (SWGDAM)^37^, the master mix has also been improved to reduce the amplification time and to cope with the PCR inhibitors. Rapid amplification of multiplex STRs for human identification has also been demonstrated by changing the master mix and/or enzyme^22,23,38,39^. There are certain multiplex kits specially designed for direct amplification of single source samples for the population data purpose. However, still most of the commonly used multiplex STR amplification kits need the pre-processing of samples before DNA amplification and genotyping^40^. Also, these specially designed kits vary in their markers. Therefore, if the standard kits are used for direct and rapid amplification, resultant profiles will not be comparable for all the tested markers with the profiles from routine casework samples and also from the existing DNA databases. Most of the widely used commercially available kits (non-direct) are not meant or reported to amplify the samples without DNA extraction. Working on a research project on X-STR’s, we collected saliva samples of unrelated individuals and used Investigator Argus X-12 multiplex STR kit (Qiagen, Germany) for DNA amplification as per manufacturer’s recommendation. DNA was isolated and extracted from 188 saliva samples using automated DNA extraction system 12 GC (Precision System Science Co., Ltd., Matsudo, Japan) following manufacturer’s instructions and pre formulated kits^41^. Few samples were tried for direct amplification of saliva samples with the same multiplex kit, using the same protocol for amplification. Promising results in terms of good quality DNA profile from the pilot study resulted into this detailed study on the direct amplification of saliva samples using commercially available non-direct multiplex kits. Kits used in the study were from leading brands including Thermo Fisher Scientific (AMPF*L*STR IDENTIFILER, AMPF*L*STR IDENTIFILER PLUS, GLOBALFILER, AMPF*L*STR Y FILER and AMPF*L*STR YFILER PLUS), Promega (POWERPLEX 16HS SYSTEM, POWERPLEX 21 SYSTEM, POWERPLEX FUSION 6C SYSTEM and POWERPLEX Y 23 SYSTEM) and Qiagen (INVESTIGATOR IDPLEX PLUS, and INVESTIGATOR ARGUS X-12 MULTIPLEX KIT) were tested for direct amplification protocol. This novel protocol was also tested using VERIFILER PLUS PCR AMPLIFICATION KIT (Thermo Fisher Scientific) and SURE ID PANGLOBAL HUMAN DNA IDENTIFICATION KIT (Health Gene Technologies) as well newly launched but still not used in routine forensic DNA typing kits. The details of multiplex kits used in this study are mentioned in Table 1. This study was designed with the aim to test and validate the direct amplification protocol using commercially available 4, 5 and 6 dyes chemistry based non-direct multiplex kits of leading brands in forensics.

**Table 1:** Comparative list of various multiplex kits.

PCR multiplex kit	Make/manufa-cturer of the multiplex kit	Dye set used in the kit	Launching year	Turnaround time for PCR amplification as per recommended protocol by the manufacturer	STR markers included in the multiplex kit							
					Markers in the multiplex	Autosomal STRs	Sex determination	SNP	Y-STRs	X-STRs	Other	Total number of markers
AMPFISTR IDENTIFILER PCR AMPLIFICATION KIT	Thermo Fisher Scientific	5 Dye	2001	Approx 150 min	D8S1179, D21S11, D7S820, CSF1PO, D3S1358, TH01, D13S317, D16S539, D2S1338, D19S433, vWA, TPOX, D18S51, Amelogenin, D5S818 and FGA	15	Amelogenin	–	–	–	–	16
AMPFISTR IDENTIFILER PLUS PCR AMPLIFICATION KIT	Thermo Fisher Scientific	5 Dye	2010	Approx 150 min	D8S1179, D21S11, D7S820, CSF1PO, D3S1358, TH01, D13S317, D16S539, D2S1338, D19S433, vWA, TPOX, D18S51, Amelogenin, D5S818 and FGA	15	Amelogenin	–	–	–	–	16
POWERPLEX 16HS SYSTEM	Promega	4 Dye	2009	Approx 60 min	D18S51, D21S11, TH01, D3S1358, Penta E, FGA, TPOX, D8S1179, vWA, CSF1PO, D16S539, D7S820, D13S317, D5S818, Penta D and Amelogenin	15	Amelogenin	–	–	–	–	16
POWERPLEX 21 SYSTEM	Promega	5 Dye	2012	Approx 90 min	D1S1656, D2S1338, D3S1358, D5S818, D6S1043, D7S820, D8S1179, D12S391, D13S317, D16S539, D18S51, D19S433, D21S11, Amelogenin, CSF1PO, FGA, Penta D, Penta E, TH01, TPOX and vWA	20	Amelogenin	–	–	–	–	21
INVESTIGATOR IDPLEX PLUS AMPLIFICATION KIT	Qiagen	5 Dye	2014	Approx 90 min	D3S1358, TH01, D21S11, D18S51, D5S818, D13S317, D7S820, D16S539, CSF1PO, vWA, D8S1179, TPOX, FGA, D2S1338, D19S433 and Amelogenin	15	Amelogenin	–	–	–	–	16
INVESTIGATOR ARGUS X-12 KIT	Qiagen	5 Dye	2015	Approx 60 min	DXS10103, DXS8378, DXS7132, DXS10134, DXS10074, DXS10101, DXS10135, DXS7423, DXS10146, DXS10079, HPRTB and DXS10148	–	Amelogenin	–	–	12	–	12
GLOBALFILER PCR AMPLIFICATION KIT	Thermo Fisher Scientific	6 Dye	2013	Approx 90 min	D3S1358, vWA, D16S539, CSF1PO, TPOX, D8S1179, D21S11, D18S51, D2S441, D19S433, TH01, FGA, D22S1045, D5S818, D13S317, D7S820, SE33, D10S1248, D1S1656, D12S391, D2S1338,DYS391,Y indel and Amelogenin	21	Amelogenin	Y Indel	1	–	–	24
SURE ID PANGLOBAL HUMAN DNA IDENTIFICATION KIT	Health Gene Technologies	6 Dye	2018	Approx 60 min	D3S1358, TH01, D21S11, D18S51, Penta E, D12S391, D6S1043, D2S1338, D1S1656 , D5S818, D13S317, D7S820, D19S433, CSF1PO, Penta D, D2S441, vWA, D8S1179, TPOX, FGA, D16S539, D22S1045, SE33, D10S1248, Alelogenin, Y Indel and DYS391	24	Amelogenin	Y Indel	1	–	–	27
POWERPLEX FUSION 6C SYSTEM	Promega	6 Dye	2015	Approx 90 min	CSF1PO, FGA, TH01, vWA, D1S1656, D2S1338, D2S441, D3S1358, D5S818, D7S820, D8S1179, D10S1248, D12S391, D13S317, D16S539, D18S51, D19S433, D21S11, Amelogenin, DYS391, Penta D, Penta E, D22S1045, TPOX, SE33, DYS570 and DYS576	23	Amelogenin	–	3	–	–	27
VERIFILER PLUS PCR AMPLIFICATION KIT	Thermo Fisher Scientific	6 Dye	2018	Approx 90 min	D3S1358, vWA, D16S539, CSF1PO, D6S1043, D8S1179, D21S11, D18S51, D5S818, D2S441, D19S433, FGA, D10S1248, D22S1045, D1S1656, D13S317, D7S820, Penta E, Penta D, TH01, D12S391, D2S1338, TPOX,IQCS and IQCL, Y indel and Amelogenin	23	Amelogenin	Y Indel	–	–	2 Quality sensor Markers	27
POWERPLEX Y 23 SYSTEM	Promega	5 Dye	2012	Approx 90 min	DYS576, DYS389I, DYS448, DYS389II, DYS19, DYS391, DYS481, DYS549, DYS533, DYS438, DYS437, DYS570, DYS635, DYS390, DYS439, DYS392, DYS643, DYS393, DYS458, DYS385a/b, DYS456 and Y-GATA-H4	–	–	–	23	–	–	23
AMPF*L*STR YFILER PCR AMPLIFICATION KIT	Thermo Fisher Scientific	5 Dye	2004	Approx 150 min	DYS456,DYS389 I, DYS390,DYS389 II, DYS458, DYS19, DYS385 a/b, DYS393,DYS391, DYS439, DYS635, DYS392, Y GATA H4, DYS437, DYS438 and DYS448	–	–	–	17	–	–	17
YFILER PLUS PCR AMPLIFICATION KIT	Thermo Fisher Scientific	6 Dye	2014	Approx 90 min	DYS576, DYS389I, DYS635, DYS389II, DYS627, DYS460, DYS458, DYS19, YGATAH4, DYS448, DYS391, DYS456, DYS390, DYS438, DYS392, DYS518, DYS570, DYS437, DYS385, DYS449, DYS393, DYS439, DYS481, DYF387S1 and DYS533	–	–	–	27	–	–	27

## Result and discussion

### Optimizing parameters for direct PCR amplification

For the pilot study, initially 10 saliva samples were directly amplified using both the recommended full reaction volume of 25 µl and a reaction mixture with reduced volume of 10 µl with the help of PCR using all the tested multiplex kits. The DNA profile obtained from both the reaction volumes was evaluated, considering amplification of all the markers to showcase a full DNA profile, peak heights including inter and intra marker balance, observation of stutter and/or other artifacts. In this preliminary test, all the tested samples produced quality DNA profiles with both the reaction volumes and showed concordance. Our few previous studies have also reported use of 10 µl PCR reaction volume for an efficient reaction product, which further supported the reduction of final PCR reaction volume^42,43^. Direct amplification protocol was further validated for 10 µl reaction volume PCR reaction as per recommended PCR conditions of the particular multiplex kits. Using the above said direct amplification protocol, all the tested samples were directly amplified using non-direct 4 dyes chemistry based autosomal STR marker multiplex kit POWERPLEX 16HS SYSTEM (now not commonly used in forensics), 5 dyes chemistry based autosomal STR marker multiplex kits AMPF*L*STR IDENTIFILER, AMPF*L*STR IDENTIFILER PLUS, POWERPLEX 21 SYSTEM and INVESTIGATOR IDPLEX PLUS, 6 dyes chemistry based autosomal STR marker multiplex kits GLOBALFILER, POWERPLEX FUSION 6C SYSTEM, VERIFILER PLUS and SURE ID PANGLOBAL HUMAN DNA IDENTIFICATION KIT; Y-STR kits AMPF*L*STR YFILER, POWERPLEX Y 23 SYSTEM and AMPF*L*STR YFILER PLUS MULTIPLEX KITS; X-STR kit INVESTIGATOR ARGUS X-12 MULTIPLEX KIT. For all the used kits, standard recommended conditions except final reaction volume were followed. Complete and quality DNA profile could be obtained with all the tested multiplex kits using this protocol.

All the tested samples were directly amplified at all the loci with the all the above mentioned multiplex PCR kits used in this study. However, it should be noted that the performance evaluation by the peak heights between the kits is only an approximation. It is because the same loci are labeled with different fluorescent dyes and have different amplicon sizes in different kits^22^ and for the proper assessment of DNA profile, the overall quality of the profile using profile quality measures is more important^44^.

In conventional PCR method, Tris EDTA (TE) buffer is used for the final volume adjustment, which has also been reported to inhibit PCR amplification to some extent^19,45^. In this study, amplification grade water was used for final volume adjustment, which reduced the chances of reaction inhibition due to TE. In the case of DNA degradation or inhibition, larger/high molecular weight loci (usually more that 300 bp loci) reflect low peak height, higher peak imbalance or allele drop outs. This effect leads to development of a DNA profile with a slope peak height, also termed as SKI slope effect^19,46^. Noticeably, in our study almost all the DNA profiles obtained were having balanced peak heights without SKI slope effect. Rarely, very few DNA profiles were observed with the SKI slope effect which was due to the degradation of the samples. This was confirmed by the usual automated extraction followed by RT PCR of those few samples (data not shown).

### Conventional DNA typing versus direct amplification methods

To determine the difference between the conventional DNA typing (including DNA extraction, quality and quantity check, amplification and Genotyping), and the present method of direct amplification, and to estimate the DNA quantity likely to be present in the direct amplification reaction, 10 saliva samples were used for amplification with both the methods. The mean quantity of DNA was found to be 0.45 ng for all the 10 tested samples and most of the multiplex kits claim to be more sensitive for more than 0.25 ng input DNA^47,48^. With conventional and direct amplification methods, all the alleles were observed at all the loci of respective multiplex kits and balanced high quality DNA profiles were obtained, thus establishing concordance. Further, all the tested samples of this study were directly amplified using commonly used non-direct multiplex kits used in forensic DNA typing (Table 1). Direct PCR amplification protocol from the reference saliva samples by omitting DNA extraction and quantification steps, which resulted in 80% reduction of the turnaround time (Table 2).

**Table 2:** Turnaround time for DNA typing process.

Turnaround time for DNA isolation					DNA quantitation		Total time required from DNA isolation to DNA quantitation (approx in minutes)		Turnaround time for PCR and genotyping
Manual	Automatic DNA extraction system				Quantitation using quantfiler/quantifiler duo kit	Quatatitation using quantfiler trio kit			
Organic extraction Method (phenol chloroform isoamyl alcohol-PCIA)	Pre-processing time		Time on machine	Total time					
							Manual	Automatic	
Approx 300 min	Approx 30–70 min		Approx 18–30 min	Approx 100 min	Approx 100 min	Approx 50 min	Approx 350/400 min	Approx 200/250 min	Approx 120–200 min
Method in practice		Effective turnaround time of complete DNA Processing up to pre-PCR (including multistep handling time) approx 80%							Around 20%
Proposed method		Not required							Remains same

### Assessment of DNA profile quality

To assure the quality of DNA profile, Total Peak Height (TPH), Peak Height Ratio (PHR), inter and intra locus balance (local and global balance) of the obtained DNA profiles were analyzed.

TPH is the sum of peak heights of both the heterozygous alleles in the DNA profile. PHR for heterozygous alleles defines the ratio of lower peak height and higher peak height at the specific marker. This value ranges from 0 to 1, where 1 represents the two identical peaks with equal heights and 0 represents the situation where one of the peaks is not observed which might be because of allele drop out due to PCR inhibition or degraded DNA^44^.

$$ {\text{PHR}} = \begin{array}{*{20}l} {{\text{Height }}\,{\text{of }}\,{\text{lower }}\,{\text{peak}}} \hfill \\ {{\text{PHR}} = \{ - - - - - - - - - - - {\text{for }}\,{\text{a}}\,{\text{ heterozygous }}\,{\text{allele }}\,{\text{at }}\,{\text{particular }}\,{\text{locus}}} \hfill \\ {{\text{Height}}\,{\text{ of}}\,{\text{ higher}}\,{\text{ peak}}} \hfill \\ {} \hfill \\ \end{array} $$

$$ {\text{PHR}} = {1 }\,{\text{for }}\,{\text{true }}\,{\text{homozygous }}\,{\text{allele }}\,{\text{at }}\,{\text{particular }}\,{\text{locus}} $$

Inter and Intra locus balance was measured and represented in the local balance and global balance respectively, which is mean of TPH for all the tested loci in the multiplex kits.

Overall TPH, PHR and global balance quality parameters of DNA profile were evaluated statistically using one way analysis of variance (ANOVA) non parametric applying Friedman test at 5 percent of significant level and Dunn's Multiple Comparison test using Prism GrapghPad v5 software^49^.

### Autosomal STR multiplex kits

#### AMPFLSTR IDENTIFILER AND AMPFLSTR IDENTIFILER PLUS PCR AMPLIFICATION KIT

**Figure 2:**
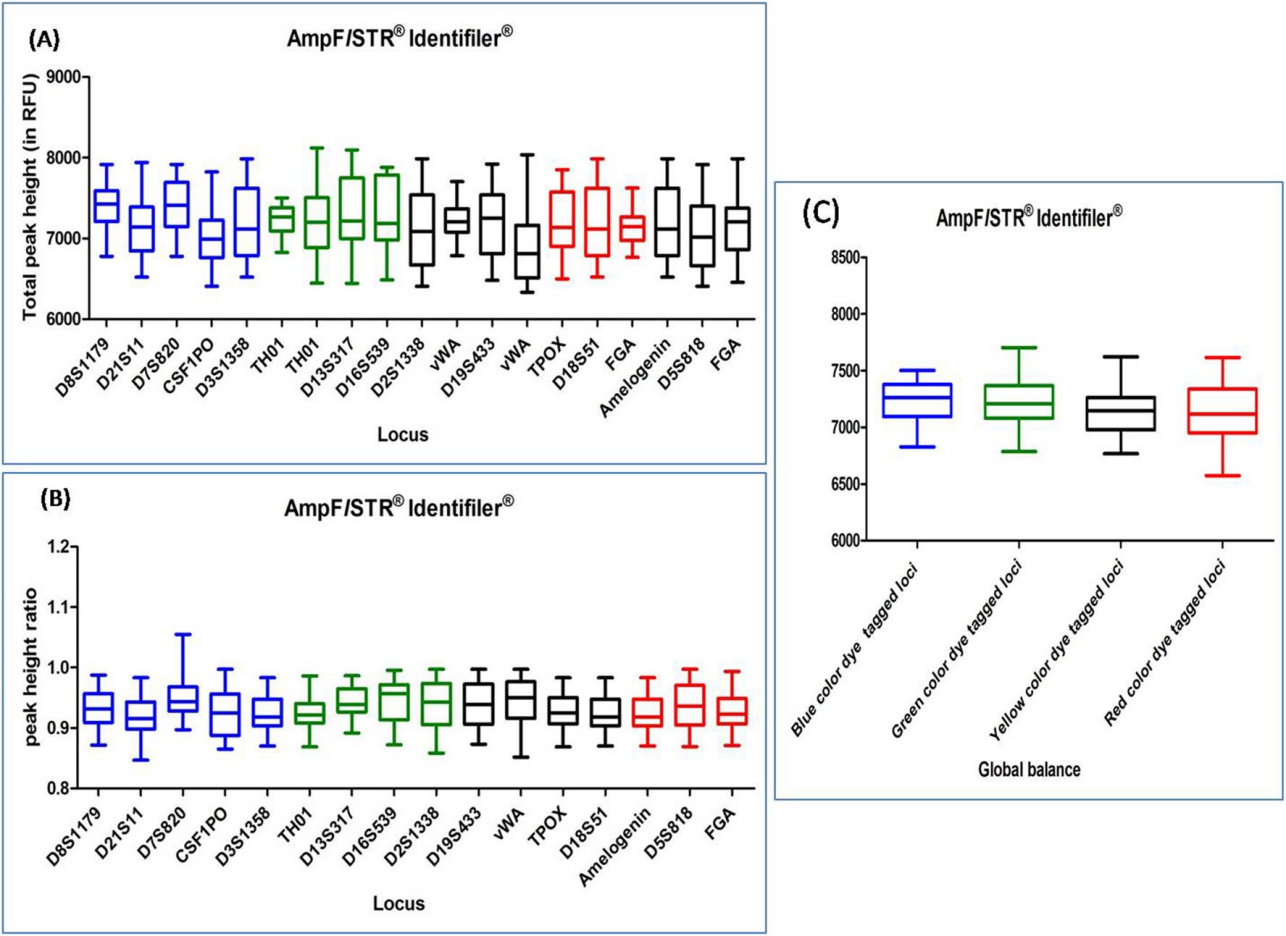
Quality assessment of DNA profile by Dunn's multiple comparison test for the AMPF*L*STR IDENTIFILER PCR AMPLIFICATION KIT (**A**) Total peak height (**B**) Peak height ratio (**C**) Global balance.

**Figure 3:**
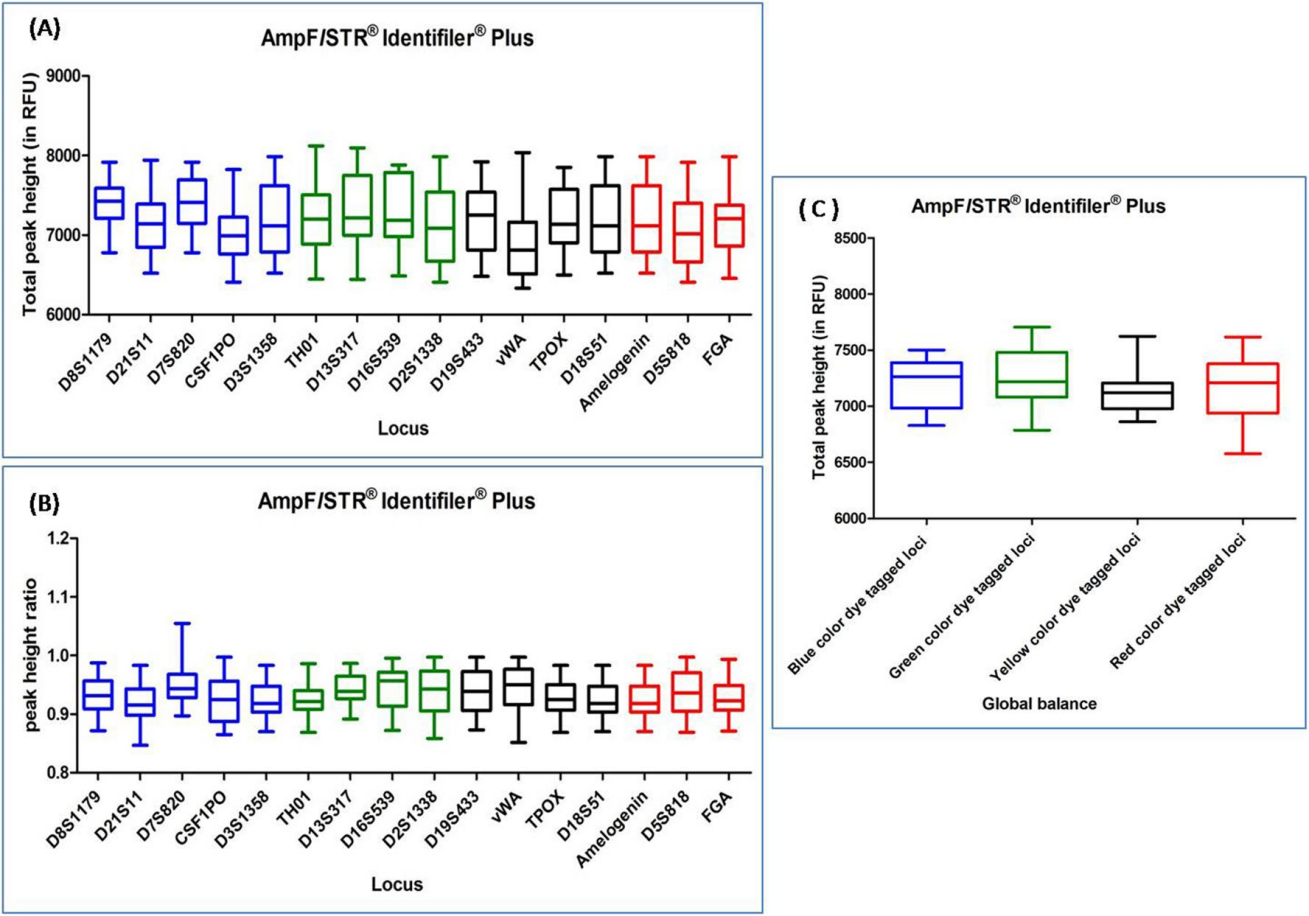
Quality assessment of DNA profile by Dunn's multiple comparison test for the AMPF*L*STR IDENTIFILER PLUS PCR AMPLIFICATION KIT (**A**) Total peak height (**B**) Peak height ratio (**C**) Global balance.

DNA profiles obtained with these multiplex kits were found to be complete and balanced. The average TPH and PHR ranged from 6553 to 7833 and from 0.875 to 0.984, respectively for both the kits. Out of 103 DNA profiles, only five DNA profiles were observed with ski slope effect. In Dunn's Multiple Comparison test, at 5 percent significant level, all the DNA profiles showed no significant variation in the global balance (Figs. 2, 3), (Supplementary Tables S1, S2).

#### POWERPLEX 16HS SYSTEM

**Figure 4:**
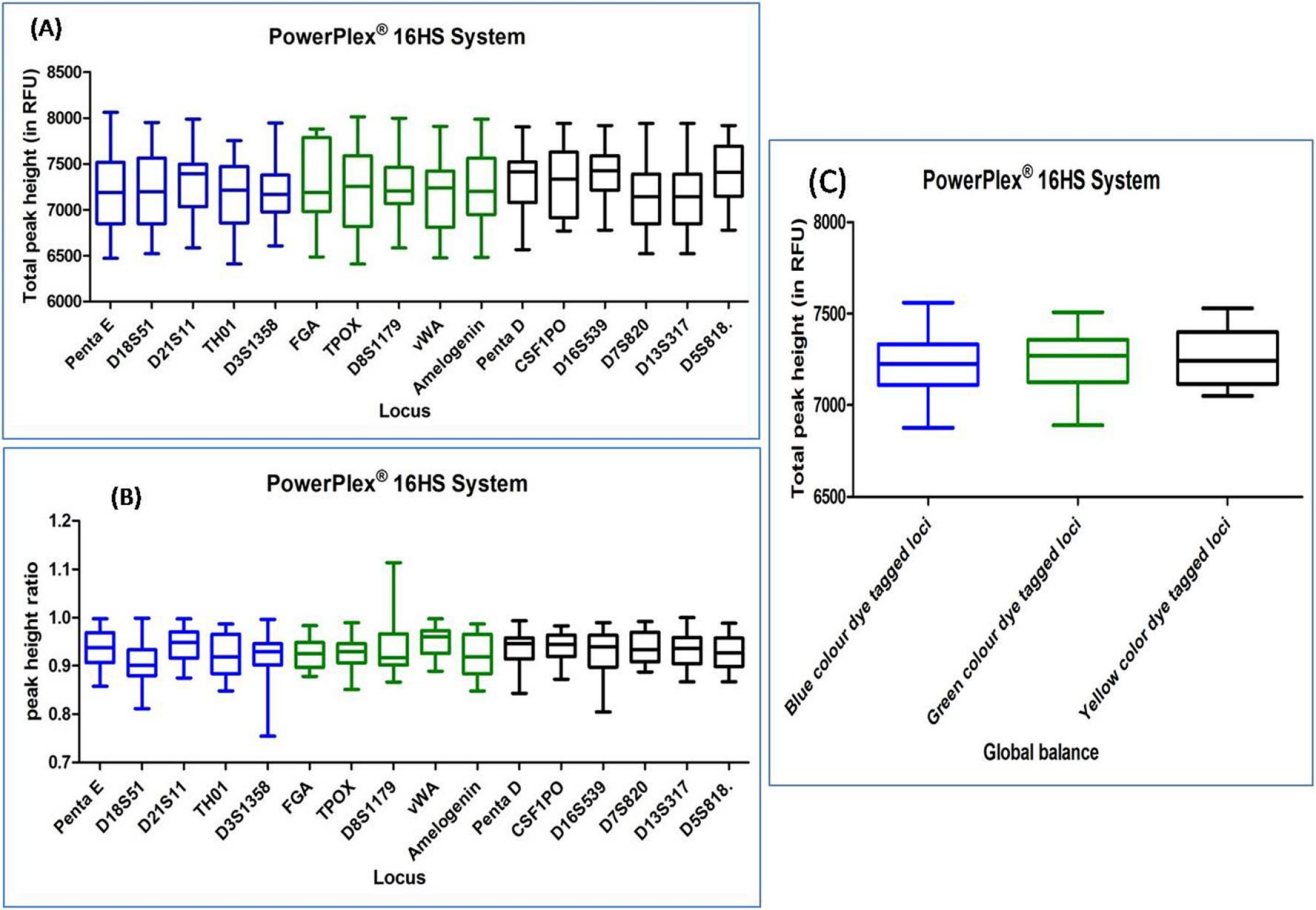
Quality assessment of DNA profile by Dunn's multiple comparison test for the POWERPLEX 16HS SYSTEM (**A**) Total peak height (**B**) Peak height ratio (**C**) Global balance.

DNA profiles obtained with this multiplex kit was also found to be balanced with an average TPH and PHR ranging from 6593 to 7889 and 0.863 to 0.993 respectively. Seven DNA profiles were observed with ski slope effect. In Dunn's Multiple Comparison test, at 5 percent significant level, all the DNA profiles exhibited no significant variation in the global balance (Fig. 4), (Supplementary Table S3).

#### POWERPLEX 21 SYSTEM

**Figure 5:**
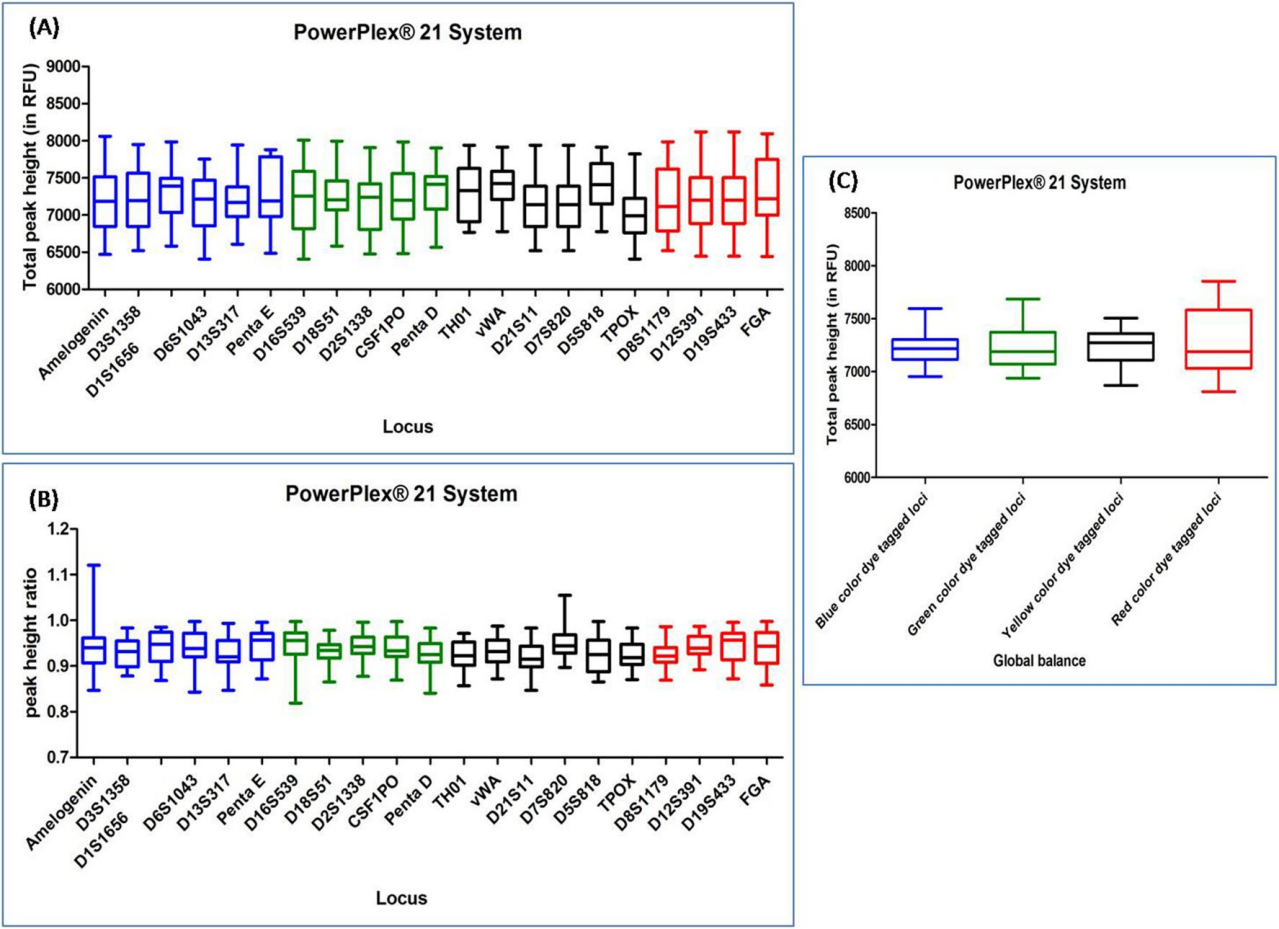
Quality assessment of DNA profile by Dunn's multiple comparison test for the POWERPLEX 21 SYSTEM (**A**) Total peak height (**B**) Peak height ratio (**C**) Global balance.

DNA profiles obtained with this multiplex kit were found to have average TPH and PHR ranging from 6548 to 7925 and from 0.865 to 0.993 respectively. Out of the total studied DNA profiles, ten DNA profiles were observed to have ski slope effect. In Dunn's Multiple Comparison test, at 5 percent significant level, all the DNA profiles accounted no significant variation in the global balance (Fig. 5), (Supplementary Table S4).

#### INVESTIGATOR IDPLEX PLUS KIT

**Figure 6:**
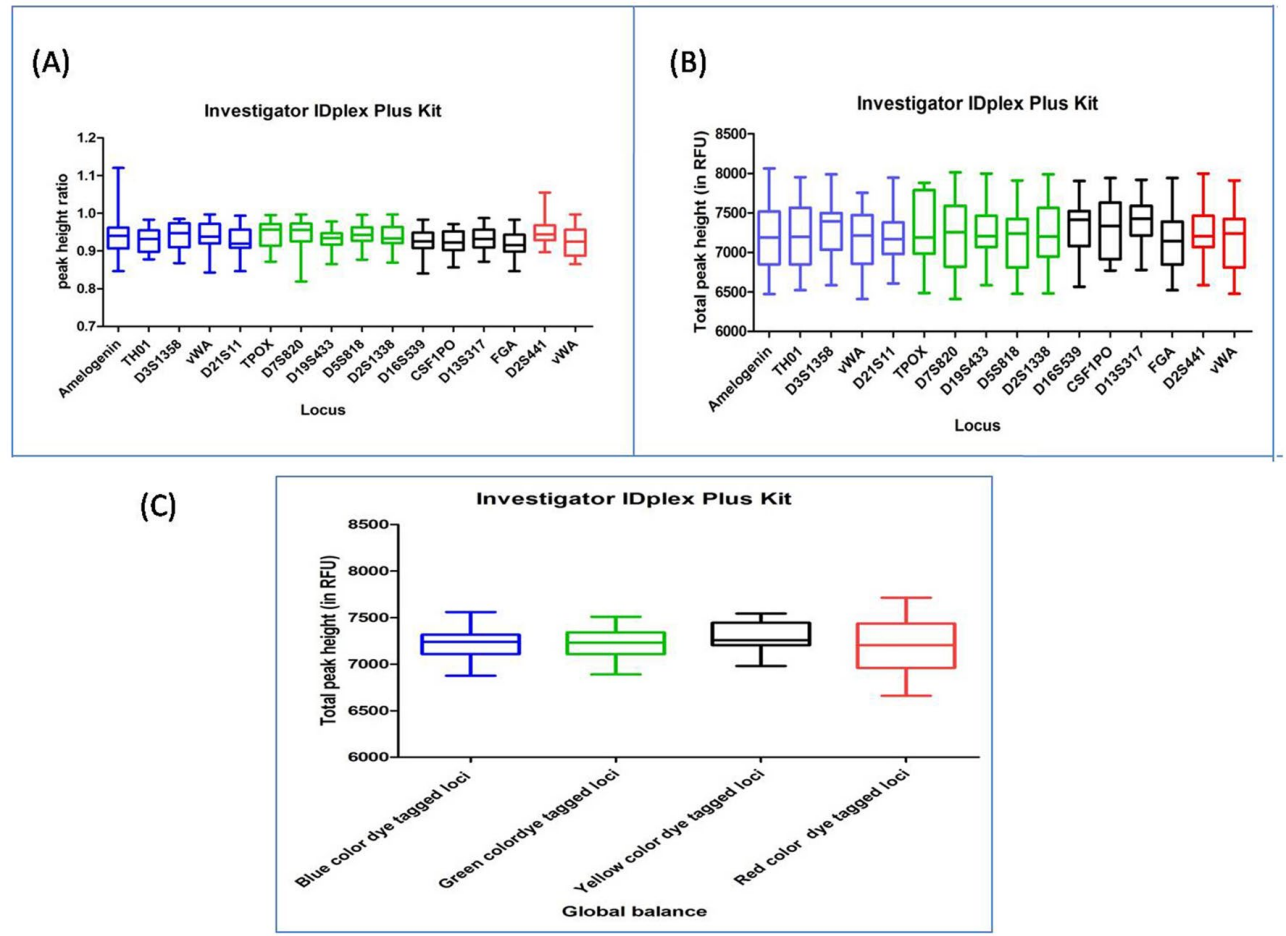
Quality assessment of DNA profile by Dunn's multiple comparison test for the INVESTIGATOR IDPLEX PLUS KIT (**A**) Total peak height (**B**) Peak height ratio (**C**) Global balance.

DNA profiles obtained with this multiplex kit were found to have an average TPH and PHR ranging from 6593 to 7883 and from 0.866 to 0.993, respectively. Out of the total studied DNA profiles, fourteen DNA profiles were observed with ski slope effect. In Dunn's Multiple Comparison test, at 5 percent significant level, all the DNA profiles exhibited no significant variation in the global balance (Fig. 6), (Supplementary Table S5).

#### POWERPLEX FUSION 6C SYSTEM

**Figure 7:**
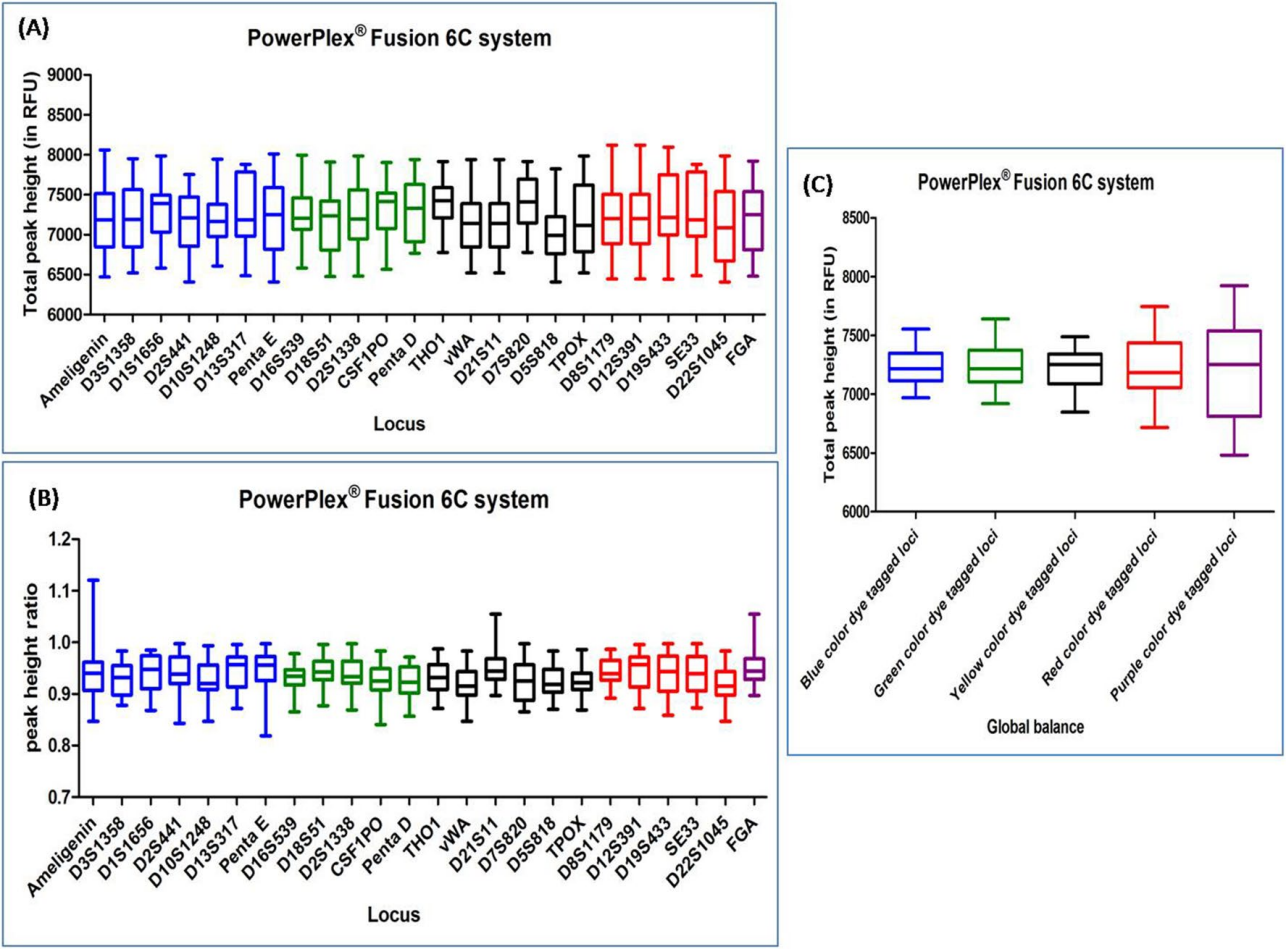
Quality assessment of DNA profile by Dunn's multiple comparison test for the POWERPLEX FUSION 6C SYSTEM (**A**) Total peak height (**B**) Peak height ratio (**C**) Global balance.

DNA profiles obtained with this multiplex kit were found to have an average TPH and PHR values ranging from 6548 to 7927 and from 0.864 to 0.994, respectively. Out of the total studied DNA profiles, ten DNA profiles were observed with ski slope effect. In Dunn's Multiple Comparison test, at 5 percent significant level, all the DNA profiles accounted no significant variation in the global balance (Fig. 7), (Supplementary Table S6).

#### GLOBALFILER PCR AMPLIFICATION KIT

**Figure 8:**
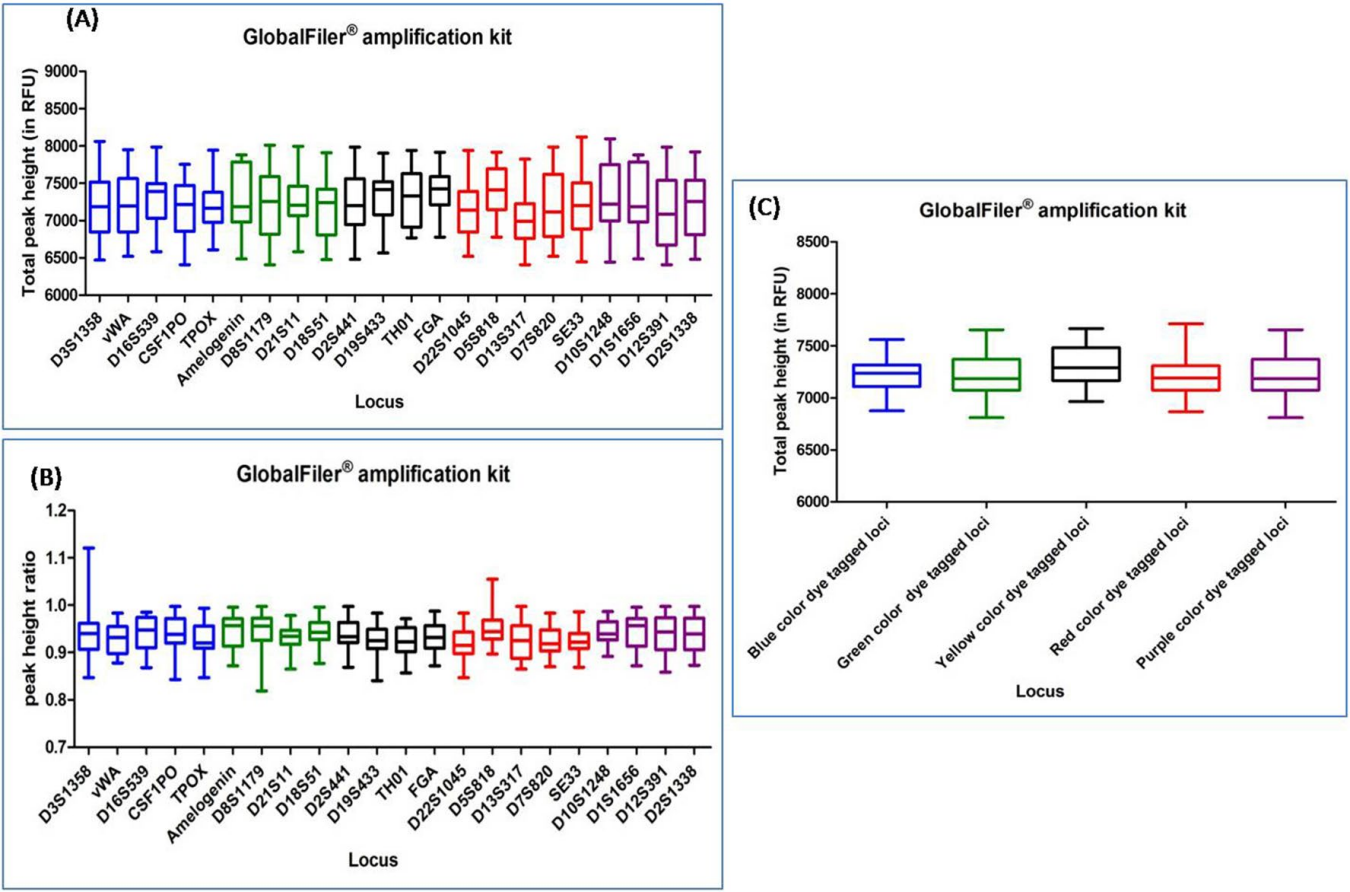
Quality assessment of DNA profile by Dunn's multiple comparison test for the GLOBALFILER PCR AMPLIFICATION KIT (**A**) Total peak height (**B**) Peak height ratio (**C**) Global balance.

DNA profiles obtained with this multiplex kit showed average TPH and PHR values ranging from 6546 to 7924 and from 0.861 to 0.994, respectively. Out of the total studied DNA profiles, ten DNA profiles showed ski slope effect. In Dunn's Multiple Comparison test, at 5 percent significant level, all the DNA profiles exhibited no significant variation in the global balance (Fig. 8), (Supplementary Table S1).

#### VERIFILER PLUS PCR AMPLIFICATION KIT

**Figure 9:**
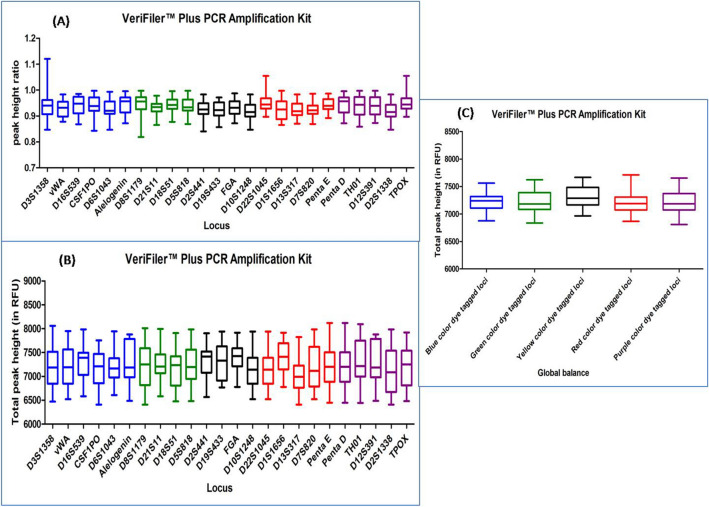
Quality assessment of DNA profile by Dunn's multiple comparison test for the VERIFILER PLUS PCR AMPLIFICATION KIT (**A**) Peak height ratio (**B**) Total peak height (**C**) Global balance.

DNA profiles obtained with this multiplex kit had an average TPH and PHR values ranging from 6549 to 7928 and from 0.867 to 0.995 respectively. Out of the total studied DNA profiles, nine DNA profiles were with ski slope effect. In Dunn's Multiple Comparison test, at 5 percent significant level, all the DNA profiles exhibited no significant variation in the global balance (Fig. 9), (Supplementary Table S8).

#### SURE ID PANGLOBAL HUMAN DNA IDENTIFICATION KIT

**Figure 10:**
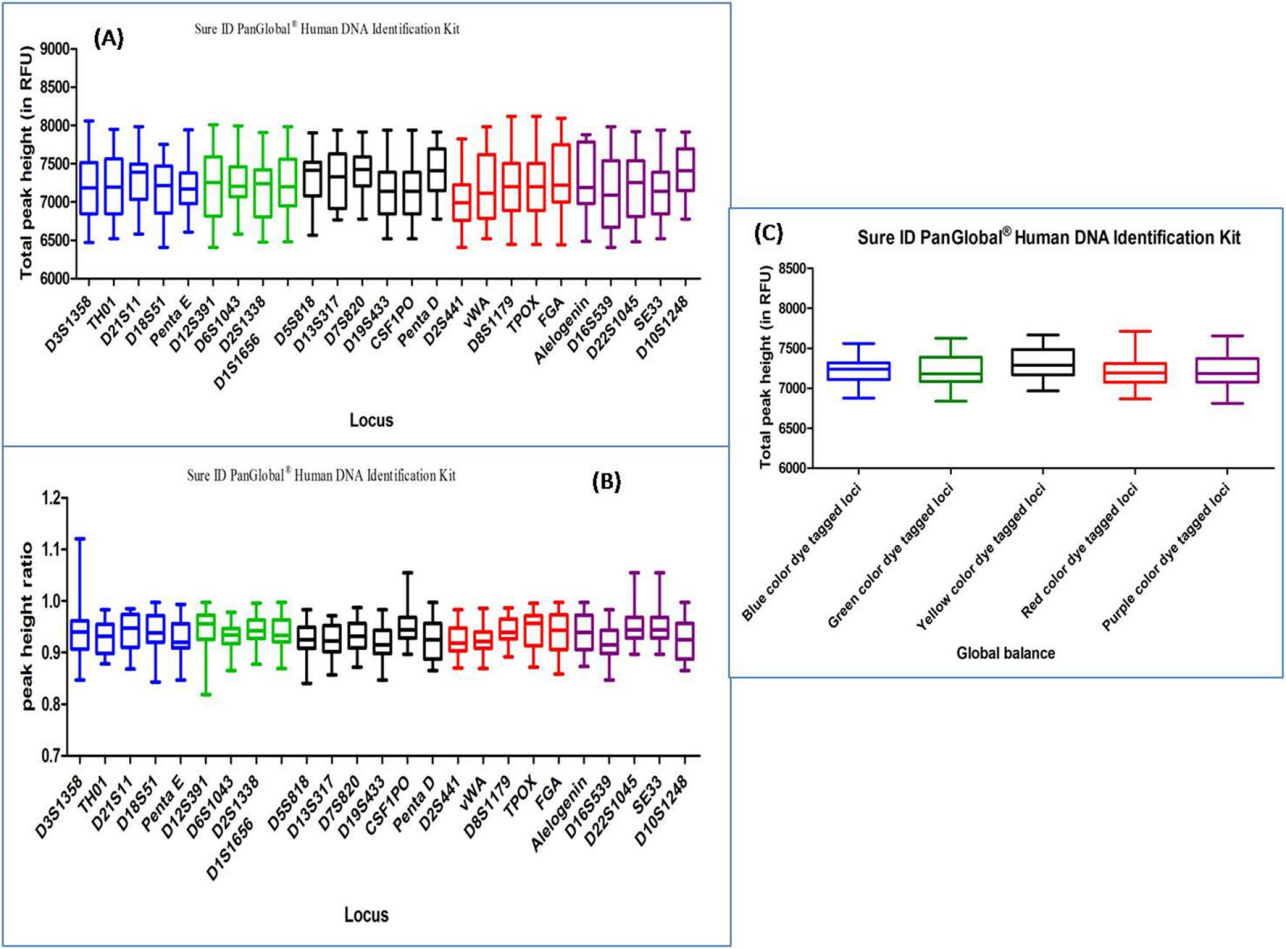
Quality assessment of DNA profile by Dunn's multiple comparison test for the SURE ID PANGLOBAL HUMAN DNA IDENTIFICATION KIT (**A**) Total peak height (**B**) Peak height ratio (**C**) Global balance.

DNA profiles obtained with this multiplex kit were found to have an average TPH and PHR values ranging from 6556 to 7922 and from 0.866 to 0.996 respectively. Out of the total studied DNA profiles, fifteen DNA profiles showed ski slope effect. In Dunn's Multiple Comparison test, at 5 percent significant level, all the DNA profiles did not exhibit any significant variation in the global balance (Fig. 10), (Supplementary Table S9).

### Y-STR multiplex kits

All the male samples in this study were directly amplified using Y STR multiplex kits. The haplotype data of these samples were statistically evaluated on TPH and global balance parameters. The obtained profiles matched the standard criterion of profile quality index for most of the tested sample.

#### POWERPLEX Y 23 SYSTEM

**Figure 11:**
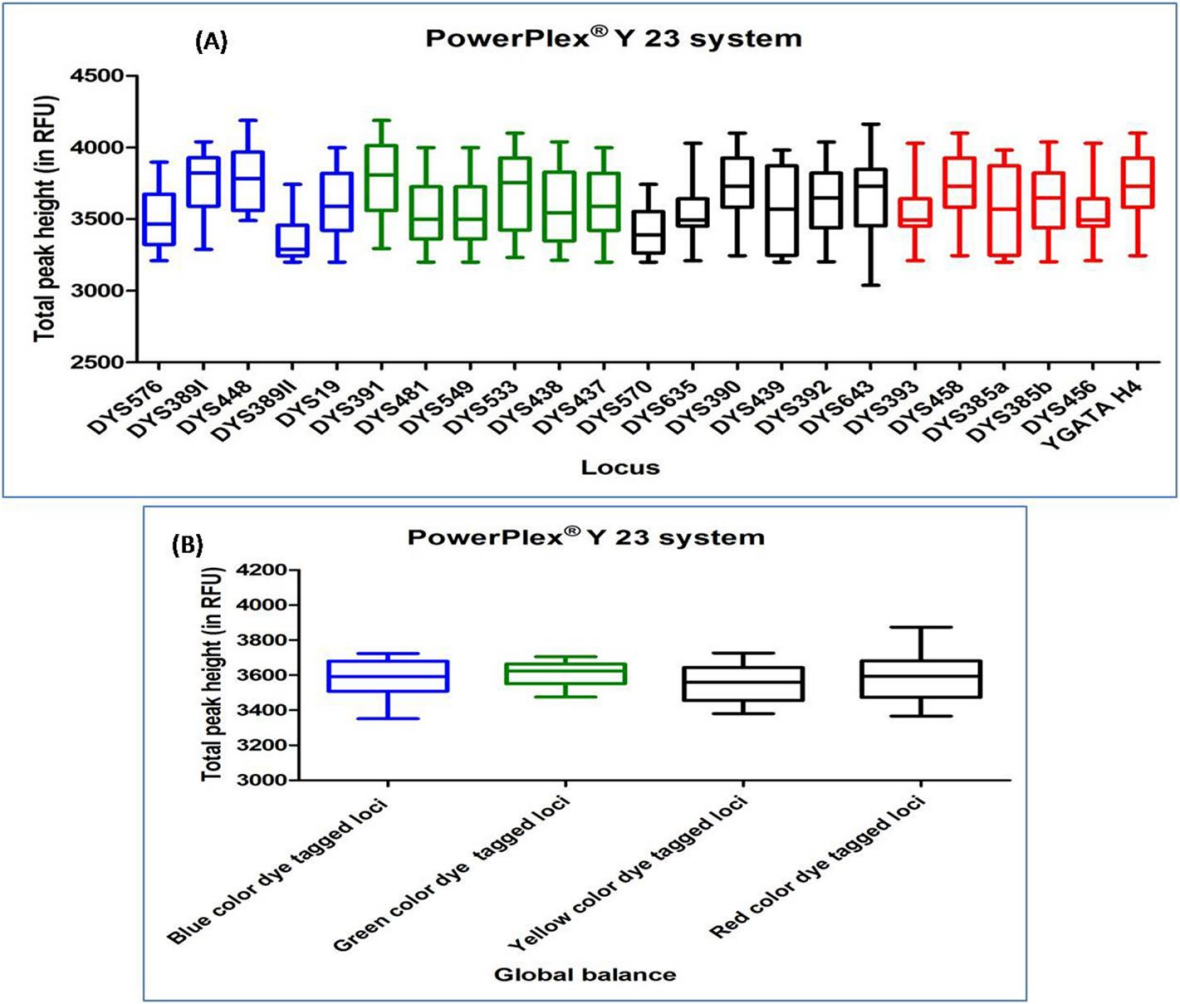
Quality assessment of DNA profile by Dunn's multiple comparison test for the POWERPLEX Y 23 SYSTEM (**A**) Total peak height (**B**) Global balance.

DNA profiles obtained with this multiplex kit showed an average TPH range from 3224 to 4019. Out of the total studied DNA profiles, six DNA profiles were observed with ski slope effect. In Dunn's Multiple Comparison test, at 5 percent significant level, all the DNA profiles accounted no significant variation in the global balance (Fig. 11), (Supplementary Table S10).

#### AMPFLSTR YFILER PCR AMPLIFICATION KIT

**Figure 12:**
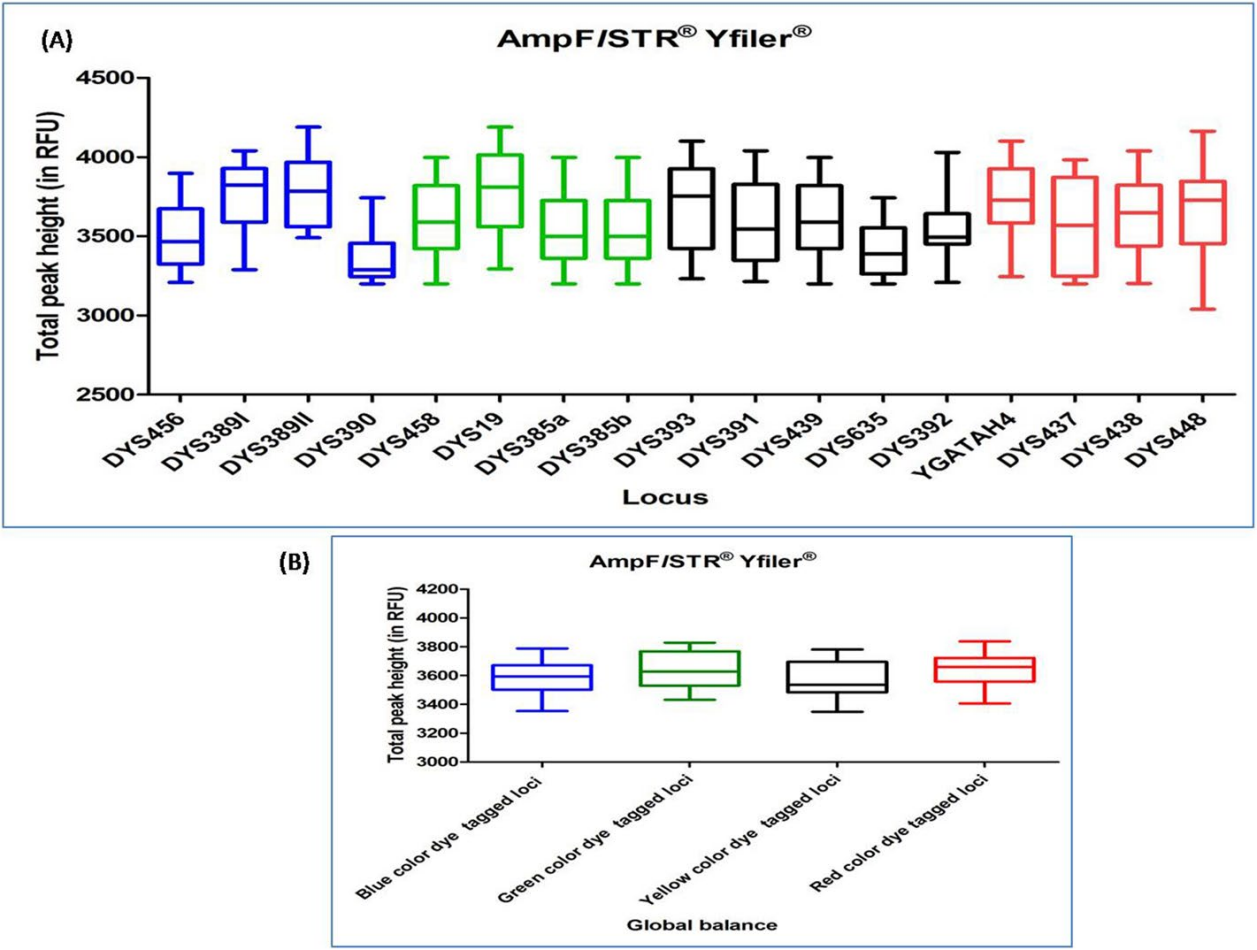
Quality assessment of DNA profile by Dunn's multiple comparison test for the AMPFLSTR YFILER PCR AMPLIFICATION KIT (**A**) Total peak height (**B**) Global balance.

DNA profiles obtained with this multiplex kit were found to have an average TPH values ranging from 3211 to 4015. Out of the total studied DNA profiles, eleven DNA profiles were observed with ski slope effect. In Dunn's Multiple Comparison test, at 5 percent significant level, all the DNA profiles did not show any significant variation in the global balance (Fig. 12), (Supplementary Table S11).

#### AMPFLSTR YFILER PLUS PCR AMPLIFICATION KIT

**Figure 13:**
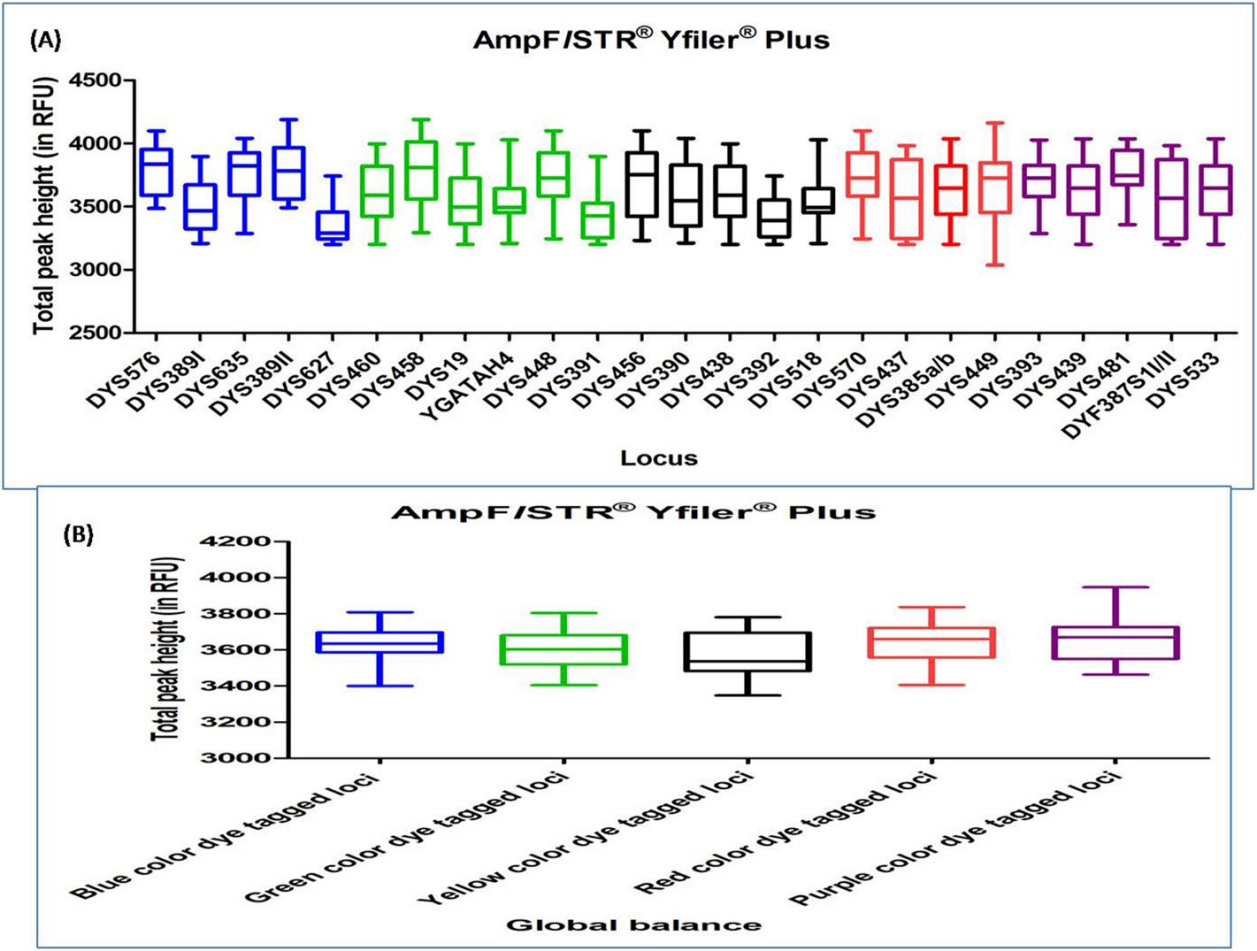
Quality assessment of DNA profile by Dunn's multiple comparison test for the AMPFLSTR YFILER PLUS PCR AMPLIFICATION KIT (**A**) Total peak height (**B**) Global balance.

DNA profiles obtained with this multiplex kit were found to have an average TPH ranging from 3213 to 4051. Out of the total studied DNA profiles, seven DNA profiles had ski slope effect. In Dunn's Multiple Comparison test, at 5 percent significant level, all the DNA profiles showed no significant variation in the global balance (Fig. 13), (Supplementary Table S12).

### X-STR multiplex kit

**Figure 14:**
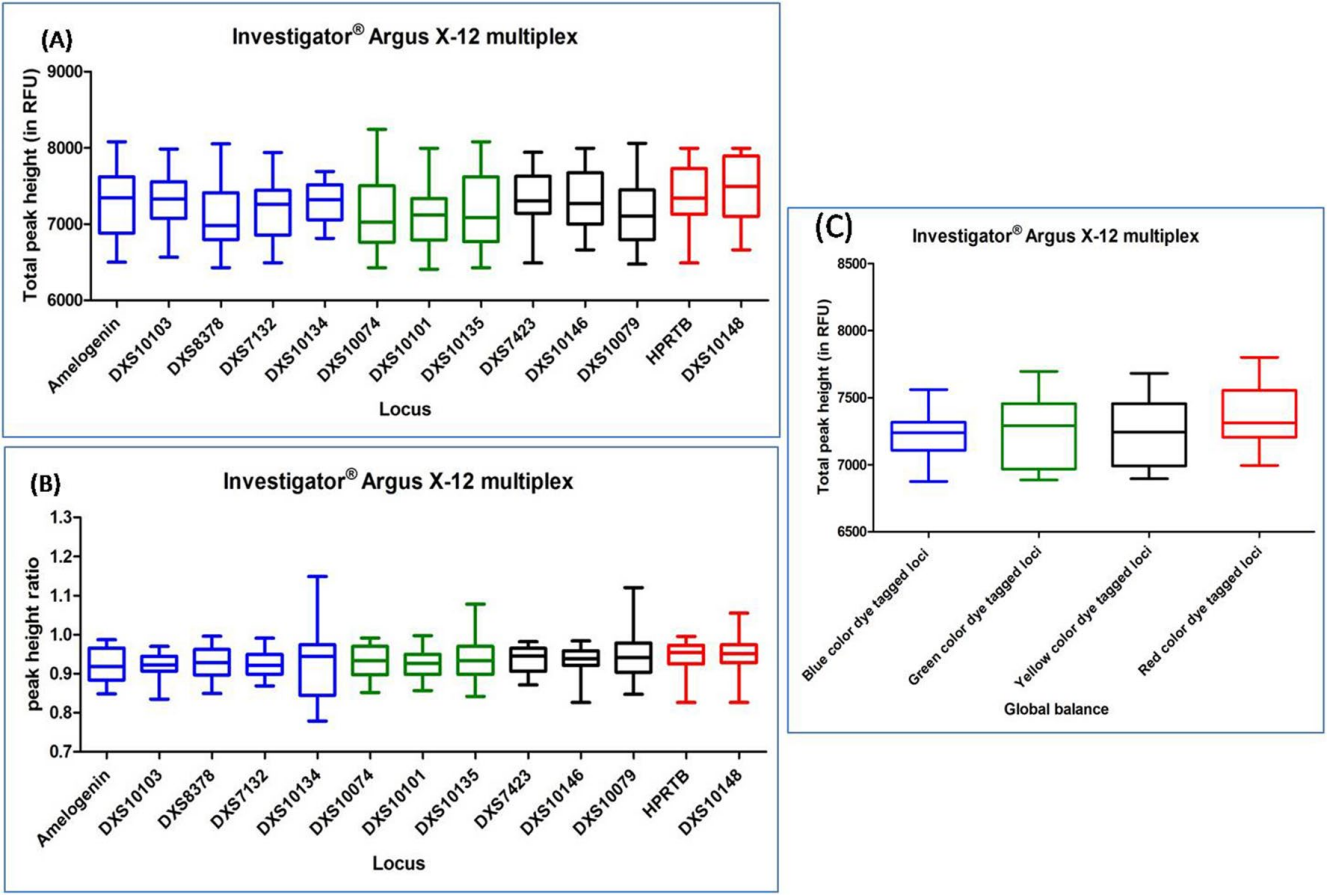
Quality assessment of DNA profile by Dunn's multiple comparison test for the INVESTIGATOR ARGUS X-12 MULTIPLEX KIT (**A**) Total peak height (**B**) Peak height ratio (**C**) Global balance.

Since male sample possesses only one X chromosome, it resulted in single peak at particular loci of X-STR marker, and female samples showed two peaks at particular loci due to the two chromosomes. Thus, the DNA profile obtained from the male samples were evaluated for TPH and global balance and the DNA profile obtained from the female samples were evaluated for TPH, PHR and global balance as well. Here we used the mean value of THP, PHR for this evaluation. Conclusive outcomes have been represented in Fig. 14 and Supplementary Table S13 for both the male and female DNA profiles accordingly.

#### INVESTIGATOR ARGUS X-12 MULTIPLEX KIT

DNA profiles obtained with this multiplex kit were found to have an average TPH value ranging from 6593 to 7949 (for both the male and female DNA profiles) and the mean PHR ranged from 0.847 to 0.999 (for female DNA profiles). Out of the total studied DNA profiles, twenty three DNA profiles were observed with ski slope effect. In Dunn's Multiple Comparison test, at 5 percent significant level, all the DNA profiles showed no significant variation in the global balance (Fig. 14), (Supplementary Table S13).

Overall data suggests that this novel protocol of direct amplification worked well with all the multiplex kits used in this study. The proposed method will not only save the effective cost, but will also curb down the turnaround time. Moreover, this novel direct amplification protocol will supposedly be useful for the speedy analysis of forensic DNA cases and ultimately will lead to the justice dissemination at the earliest.

### Quality control

All the authors have passed proficiency test and quality control exercise for DNA fingerprinting from GITAD, Spain (http://gitad.ugr.es/principal.htm). Also internal laboratory control standards were followed and controls provided with multiplex kits were used.

## Methods

### Sample collection

103 saliva samples from anonymous donors, (50 males and 53 females) who hailed from the Ratlam district of Madhya Pradesh were collected in pre-sterile collection tubes. Written informed consent was taken from the subjects in compliance with the Declaration of Helsinki^50^. The above said population has already been depicted in our earlier publication, in which the authors typed the same samples for 12 XSTRs^41^ and 15 autosomal STRs^51^. All methods were carried out in accordance with the relevant guidelines and regulations. All the experimental protocols were approved by the Institutional committee of DNA Fingerprinting Unit, State Forensic Science Laboratory, Sagar, MP, India. The samples were brought to the laboratory at 4 °C and were stored at − 20 °C. Saliva samples processed with direct amplification protocol were kept in the laboratory at − 20 °C until final processing. A maximum of one month old samples were processed for the direct amplification.

### Polymerase chain reaction (PCR) and capillary electrophoresis (CE) conditions

103 saliva samples were used directly for PCR using the following multiplex systems to test the efficacy of commonly used multiplex systems viz. AMPF*L*STR IDENTIFILER, AMPF*L*STR IDENTIFILER PLUS, POWERPLEX 21 SYSTEM, INVESTIGATOR IDPLEX PLUS, GLOBALFILER, POWERPLEX FUSION 6C SYSTEM, VERIFILER PLUS, SURE ID PANGLOBAL HUMAN DNA IDENTIFICATION KIT, AMPF*L*STR YFILER, POWERPLEX Y 23 SYSTEM, AMPF*L*STR YFILER PLUS and INVESTIGATOR ARGUS X-12 MULTIPLEX KITS available for forensic and population genetic purpose. The modifications in the reaction volume, cycle number, and input saliva samples for the respective kits have been shown in Tables 3 and 4.

**Table 3:** Validated parameters of novel direct amplification protocol.

S. no.	PCR MULTIPLEX KIT	Number of samples used in the study	Final PCR volume used in the study (in µL)	Input volume of saliva sample (in µL)	Modification in PCR cycle number form the recommended by the respective multiplex system	Complete DNA profile obtained	No. of DNA profile with SKI slope	% of balanced DNA profile obtained from direct amplification protocol	Time after sample collection
1	AMPFISTR IDENTIFILER PCR AMPLIFICATION KIT	103	10	1	Two cycle increase	103	5	95	Immediately to more than one month storage of sample
2	AMPFISTR IDENTIFILER PLUS PCR AMPLIFICATION KIT	103	10	1	Two cycle increase	103	5	95	
3	POWERPLEX 16HS SYSTEM	103	10	1	One cycle decrease	103	7	93	
4	POWERPLEX 21 SYSTEM	103	10	0.5	Two cycle decrease	103	10	90	
5	INVESTIGATOR IDPLEX PLUS AMPLIFICATION KIT	103	10	1	Two cycle decrease	103	14	86	
6	INVESTIGATOR ARGUS X-12 KIT	103	10	0.5	One cycle decrease	103	23	78	
7	GLOBALFILER PCR AMPLIFICATION KIT	103	10	1	One cycle decrease	103	10	90	
8	SURE ID PANGLOBAL HUMAN DNA IDENTIFICATION KIT	103	10	1	No change in PCR cycle	103	15	85	
9	POWERPLEX FUSION 6C SYSTEM	103	10	0.5	Two cycle decrease	103	10	90	
10	VERIFILE PLUS PCR AMPLIFICATION KIT	103	10	1	No change in PCR cycle	103	9	91	
11	POWERPLEX Y 23 SYSTEM	50	10	0.5	Two cycle decrease	50	6	88	
12	AMPF*L*STR YFILER PCR AMPLIFICATION KIT	50	10	1	One cycle increase	50	11	78	
13	YFILER PLUS PCR AMPLIFICATION KIT	50	10	1	No change in PCR cycle	50	7	86

**Table 4:** Details of recommended and reduced PCR reaction volume.

Multiplex kit	PCR reaction volume recommended by the manufacturer					Reduced PCR reaction volume for the direct amplification				
	Master mix (in µL)	Primer set (volume in µL)	Sample input (containing DNA 500 pg to 1 ng) (in µL)	Amplification grade water to achieve final reaction volume (in µL)	Final reaction volume (in µL)	Master mix (in µL)	Primer set (in µL)	Sample input (in µL)	Amplification grade water to achieve final reaction volume (in µL)	Final reaction volume (in µL)
AMPFISTR IDENTIFILER PCR AMPLIFICATION KIT	10.5 + (0.5 AmpliTaq Gold DNA Polymerase)	5.5	1	7.5	25	5 + (0.2 AmpliTaq Gold DNA Polymerase)	2	1	1.8	10
AMPFISTR IDENTIFILER PLUS PCR AMPLIFICATION KIT	10	5	1	9	25	5	2	1	2	10
POWERPLEX 16HS SYSTEM	5	2.5	1	16.5	25	2.25	1.15	1	5.6	10
POWERPLEX 21 SYSTEM	5	5	1	14	25	2	2	0.5	5.5	10
INVESTIGATOR IDPLEX PLUS AMPLIFICATION KIT	7.5	2.5	1	14	25	3	1	1	5	10
INVESTIGATOR ARGUS X-12 KIT	5 + (0.6 Multi Taq2 polymerase)	2.5	1	15.9	25	2 + (0.2 Multi Taq2 polymerase)	1	0.5	5.3	10
GLOBALFILER PCR AMPLIFICATION KIT	7.5	2.5	1	14	25	3	1	1	5	10
SURE ID PANGLOBAL HUMAN DNA IDENTIFICATION KIT	7.5	2.5	1	14	25	3	1	1	5	10
POWERPLEX FUSION 6C SYSTEM	5	5	1	14	25	2	2	0.5	5.5	10
VERIFILE PLUS PCR AMPLIFICATION KIT	5	2.5	1	16.5	25	2	1	1	6	10
POWERPLEX Y 23 SYSTEM	5	2.5	1	16.5	25	2	1	0.5	5.5	10
AMPF*L*STR YFILER PCR AMPLIFICATION KIT	9.2 + (0.8 AmpliTaq Gold DNA Polymerase)	5	1	9	25	4 + (0.2 AmpliTaq Gold DNA Polymerase)	1.25	1	5.55	10
YFILER PLUS PCR AMPLIFICATION KIT	10	5	1	9	25	3	1	1	5	10

### Capillary electrophoresis

In all the cases, 0.3 µl of the total PCR product was run on an Applied Biosystems 3100/3500XL Genetic Analyzer using standard parameters and with the size standards/allelic ladder provided with the respective non-direct multiplex kit and a 10 seconds injection time. The obtained data was analyzed using GeneMapper V3.5/GeneMapper ID version 1.4 softwares (Applied Biosystems, Foster City, CA). All the amplifications were performed using ABI thermal cycler 9700 (Applied Biosystems, Foster City, CA), with the modifications (as mentioned with the respective multiplex kits). Alleles were designated on the basis of number of allele repeats with the help of allelic ladder provided along with the respective multiplex kits. Peak detection threshold was set to 50 RFUs for the allele designation.

### Statistical evaluation

To evaluate the quality of DNA profile, the Total Peak Height (TPH), Peak Height Ratio (PHR) and global balance parameters were considered. The overall DNA profile quality of direct amplification method was statistically examined applying ANOVA non parametric by Friedman test at 5 percent of significant level Dunn's Multiple Comparison test using Prism GrapghPad v5 software^49^.

## Supplementary Information

Supplementary Information.
