# Supplementary material for: Direct PCR amplification from saliva sample using non-direct multiplex STR kits for forensic DNA typing
Source: Sci Rep. 2021 Mar 29;11:7112. doi: 10.1038/s41598-021-86633-0 (PMC8007628; doi:10.1038/s41598-021-86633-0)
Supplement: Supplementary file 1 — Supplementary Information. [file 41598_2021_86633_MOESM1_ESM.docx]

**Direct PCR amplification from saliva sample using non-direct multiplex STR kits for**

**Forensic DNA typing**

Pankaj Shrivastava^1^*, Toshi Jain^1^ and R.K. Kumawat^2^

^1^DNA Fingerprinting Unit, State Forensic Science Laboratory, Sagar-470001 (MP) India

^2^DNA division, State Forensic Science Laboratory, Rajasthan, Jaipur-302016, India

*corresponding author e mail- pankaj.shrivastava@rediffmail.com

| Table S1: One way ANOVA (nonparameteric) for mean total peak heights of blue, green, yellow and red dye taged loci of AMPFLSTR IDENTIFILER MULTIPLEX KIT | | | |
| --- | --- | --- | --- |
| Friedman test |  |  |  |
| P value | 0.2168 |  |  |
| Exact or approximate P value? | Gaussian Approximation |  |  |
| P value summary | ns |  |  |
| Are means signif. different? (P < 0.05) | No |  |  |
| Number of groups | 4 |  |  |
| Friedman statistic | 4.45 |  |  |
|  |  |  |  |
| Dunn's Multiple Comparison Test | Difference in rank sum | Significant? P < 0.05? | Summary |
| Blue color dye  tagged loci  vs Green colordye tagged loci | -3 | No | ns |
| Blue color dye  tagged loci  vs Yellow color dye tagged loci | 13 | No | ns |
| Blue color dye  tagged loci  vs Red color dye tagged loci | 10 | No | ns |
| Green colordye tagged loci vs Yellow color dye tagged loci | 16 | No | ns |
| Green colordye tagged loci vs Red color dye tagged loci | 13 | No | ns |
| Yellow color dye tagged loci vs Red color dye tagged loci | -3 | No | ns |
| ns: non significant | | | |

Contact- +91 9424371946

| Table S2: One way ANOVA (nonparameteric) for mean total peak heights of blue, green, yellow and red dye taged loci of AMPFLSTR IDENTIFILER PLUS MULTIPLEX KIT | | | |
| --- | --- | --- | --- |
| Friedman test |  |  |  |
| P value | 0.2168 |  |  |
| Exact or approximate P value? | Gaussian Approximation |  |  |
| P value summary | ns |  |  |
| Are means signif. different? (P < 0.05) | No |  |  |
| Number of groups | 4 |  |  |
| Friedman statistic | 4.45 |  |  |
|  |  |  |  |
| Dunn's Multiple Comparison Test | Difference in rank sum | Significant? P < 0.05? | Summary |
| Blue color dye tagged loci  vs Green color dye tagged loci | -3 | No | ns |
| Blue color dye tagged loci  vs Yellow colordye tagged loci | 13 | No | ns |
| Blue color dye tagged loci  vs Red color dye tagged loci | 10 | No | ns |
| Green color dye tagged loci vs Yellow colordye tagged loci | 16 | No | ns |
| Green color dye tagged loci vs Red color dye tagged loci | 13 | No | ns |
| Yellow colordye tagged loci vs Red color dye tagged loci | -3 | No | ns |
| ns: non significant | | | |

| Table S3: One way ANOVA (nonparameteric) for mean total peak heights of blue, green and yellow dye taged loci of POWERPLEX 16HS MULTIPLEX KIT | | | |
| --- | --- | --- | --- |
| Friedman test |  |  |  |
| P value | 0.1667 |  |  |
| Exact or approximate P value? | Gaussian Approximation |  |  |
| P value summary | ns |  |  |
| Are means signif. different? (P < 0.05) | No |  |  |
| Number of groups | 3 |  |  |
| Friedman statistic | 3.583 |  |  |
|  |  |  |  |
| Dunn's Multiple Comparison Test | Difference in rank sum | Significant? P < 0.05? | Summary |
| Blue colour dye tagged loci vs Green colour dye tagged loci | -8 | No | ns |
| Blue colour dye tagged loci vs Yellow color dye tagged loci | -13 | No | ns |
| Green colour dye tagged loci vs Yellow color dye tagged loci | -5 | No | ns |
| ns: non significant | | | |

| Table S4: One way ANOVA (nonparameteric) for mean total peak heights of blue, green, yellow and red dye taged loci of POWERPLEX 21 SYSTEM MULTIPLEX KIT | | | |
| --- | --- | --- | --- |
| Friedman test |  |  |  |
| P value | 0.5419 |  |  |
| Exact or approximate P value? | Gaussian Approximation |  |  |
| P value summary | ns |  |  |
| Are means signif. different? (P < 0.05) | No |  |  |
| Number of groups | 4 |  |  |
| Friedman statistic | 2.15 |  |  |
|  |  |  |  |
| Dunn's Multiple Comparison Test | Difference in rank sum | Significant? P < 0.05? | Summary |
| Blue color dye tagged loci vs Green color dye tagged loci | 0 | No | ns |
| Blue color dye tagged loci vs Yellow color dye tagged loci | -11 | No | ns |
| Blue color dye tagged loci vs Red color dye tagged loci | -1 | No | ns |
| Green color dye tagged loci vs Yellow color dye tagged loci | -11 | No | ns |
| Green color dye tagged loci vs Red color dye tagged loci | -1 | No | ns |
| Yellow color dye tagged loci vs Red color dye tagged loci | 10 | No | ns |
| ns: non significant | | | |

| Table S5: One way ANOVA (nonparameteric) for mean total peak heights of blue, green, yellow and red dye taged loci of INVESTIGATOR IDPLEX PLUS MULTIPLEX KIT | | | |
| --- | --- | --- | --- |
| Friedman test |  |  |  |
| P value | 0.3691 |  |  |
| Exact or approximate P value? | Gaussian Approximation |  |  |
| P value summary | ns |  |  |
| Are means signif. different? (P < 0.05) | No |  |  |
| Number of groups | 4 |  |  |
| Friedman statistic | 3.15 |  |  |
|  |  |  |  |
| Dunn's Multiple Comparison Test | Difference in rank sum | Significant? P < 0.05? | Summary |
| Blue color dye tagged loci  vs Green colordye tagged loci | -11 | No | ns |
| Blue color dye tagged loci  vs Yellow color dye tagged loci | -15 | No | ns |
| Blue color dye tagged loci  vs Red color  dye tagged loci | -6 | No | ns |
| Green colordye tagged loci vs Yellow color dye tagged loci | -4 | No | ns |
| Green colordye tagged loci vs Red color  dye tagged loci | 5 | No | ns |
| Yellow color dye tagged loci vs Red color  dye tagged loci | 9 | No | ns |
| ns: non significant | | | |

| Table S6: One way ANOVA (nonparameteric) for mean total peak heights of blue, green, yellow, Red and purple dye taged loci of POWERPLEX FUSION 6C SYSTEM MULTIPLEX KIT | | | | | | |  |
| --- | --- | --- | --- | --- | --- | --- | --- |
| Friedman test |  | |  | |  | |  |
| P value | 0.9967 | |  | |  | |  |
| Exact or approximate P value? | Gaussian Approximation | |  | |  | |  |
| P value summary | ns | |  | |  | |  |
| Are means signif. different? (P < 0.05) | No | |  | |  | |  |
| Number of groups | 5 | |  | |  | |  |
| Friedman statistic | 0.1667 | |  | |  | |  |
|  |  | |  | |  | |  |
| Dunn's Multiple Comparison Test | Difference in rank sum | | Significant? P < 0.05? | | Summary | |  |
| Blue color dye tagged loci vs Green color dye tagged loci | 1 | | No | | ns | |  |
| Blue color dye tagged loci vs Yellow color dye tagged loci | 3 | | No | | ns | |  |
| Blue color dye tagged loci vs Red color dye tagged loci | 2 | | No | | ns | |  |
| Blue color dye tagged loci vs Purple color dye tagged loci | -1 | | No | | ns | |  |
| Green color dye tagged loci vs Yellow color dye tagged loci | 2 | | No | | ns | |  |
| Green color dye tagged loci vs Red color dye tagged loci | 1 | | No | | ns | |  |
| Green color dye tagged loci vs Purple color dye tagged loci | -2 | | No | | ns | |  |
| Yellow color dye tagged loci vs Red color dye tagged loci | -1 | | No | | ns | |  |
| Yellow color dye tagged loci vs Purple color dye tagged loci | -4 | | No | | ns | |  |
| Red color dye tagged loci vs Purple color dye tagged loci | -3 | | No | | ns | |  |
| ns: non significant | | | | | | |  |
| Table S7: One way ANOVA (nonparameteric) for mean total peak heights of blue, green, yellow, Red and Purple dye taged loci of GLOBALFILER MULTIPLEX KIT | | | | | | |  |
| Friedman test | |  | |  | |  | |
| P value | | 0.9327 | |  | |  | |
| Exact or approximate P value? | | Gaussian Approximation | |  | |  | |
| P value summary | | ns | |  | |  | |
| Are means signif. different? (P < 0.05) | | No | |  | |  | |
| Number of groups | | 5 | |  | |  | |
| Friedman statistic | | 0.8421 | |  | |  | |
|  | |  | |  | |  | |
| Dunn's Multiple Comparison Test | | Difference in rank sum | | Significant? P < 0.05? | | Summary | |
| Blue color dye tagged loci vs Green color  dye tagged loci | | 5 | | No | | ns | |
| Blue color dye tagged loci vs Yellow color dye tagged loci | | -3 | | No | | ns | |
| Blue color dye tagged loci vs Red color dye tagged loci | | 3 | | No | | ns | |
| Blue color dye tagged loci vs Purple color dye tagged loci | | 5 | | No | | ns | |
| Green color  dye tagged loci vs Yellow color dye tagged loci | | -8 | | No | | ns | |
| Green color  dye tagged loci vs Red color dye tagged loci | | -2 | | No | | ns | |
| Green color  dye tagged loci vs Purple color dye tagged loci | | 0 | | No | | ns | |
| Yellow color dye tagged loci vs Red color dye tagged loci | | 6 | | No | | ns | |
| Yellow color dye tagged loci vs Purple color dye tagged loci | | 8 | | No | | ns | |
| Red color dye tagged loci vs Purple color dye tagged loci | | 2 | | No | | ns | |
| ns: non significant | | | | | | | |

| Table S8: One way ANOVA (nonparameteric) for mean total peak heights of blue, green, yellow, Red and Purple dye taged loci of VERIFILER PLUS MULTIPLEX KIT | | | | | | | |
| --- | --- | --- | --- | --- | --- | --- | --- |
| Friedman test |  | |  | | |  | |
| P value | 0.8325 | |  | | |  | |
| Exact or approximate P value? | Gaussian Approximation | |  | | |  | |
| P value summary | ns | |  | | |  | |
| Are means signif. different? (P < 0.05) | No | |  | | |  | |
| Number of groups | 5 | |  | | |  | |
| Friedman statistic | 1.467 | |  | | |  | |
|  |  | |  | | |  | |
| Dunn's Multiple Comparison Test | Difference in rank sum | | Significant? P < 0.05? | | | Summary | |
| Blue color dye tagged loci vs Green color dye tagged loci | 4 | | No | | | ns | |
| Blue color dye tagged loci vs Yellow color dye tagged loci | -8 | | No | | | ns | |
| Blue color dye tagged loci vs Red color dye tagged loci | 2 | | No | | | ns | |
| Blue color dye tagged loci vs Purple color dye tagged loci | 2 | | No | | | ns | |
| Green color dye tagged loci vs Yellow color dye tagged loci | -12 | | No | | | ns | |
| Green color dye tagged loci vs Red color dye tagged loci | -2 | | No | | | ns | |
| Green color dye tagged loci vs Purple color dye tagged loci | -2 | | No | | | ns | |
| Yellow color dye tagged loci vs Red color dye tagged loci | 10 | | No | | | ns | |
| Yellow color dye tagged loci vs Purple color dye tagged loci | 10 | | No | | | ns | |
| Red color dye tagged loci vs Purple color dye tagged loci | 0 | | No | | | ns | |
| ns: non significant | | | | | | | |
| Table S9: One way ANOVA (nonparameteric) for mean total peak heights of blue, green, yellow, Red and Purple dye taged loci of SURE ID PANGLOBAL HUMAN DNA IDENTIFICATION KIT | | | | | | | |
| Friedman test | |  | |  |  | |  |
| P value | | 0.8325 | |  |  | |  |
| Exact or approximate P value? | | Gaussian Approximation | |  |  | |  |
| P value summary | | ns | |  |  | |  |
| Are means signif. different? (P < 0.05) | | No | |  |  | |  |
| Number of groups | | 5 | |  |  | |  |
| Friedman statistic | | 1.467 | |  |  | |  |
|  | |  | |  |  | |  |
| Dunn's Multiple Comparison Test | | Difference in rank sum | | Significant? P < 0.05? | Summary | |  |
| Blue color dye tagged loci vs Green color dye tagged loci | | 4 | | No | ns | |  |
| Blue color dye tagged loci vs Yellow color dye tagged loci | | -8 | | No | ns | |  |
| Blue color dye tagged loci vs Red color dye tagged loci | | 2 | | No | ns | |  |
| Blue color dye tagged loci vs Purple color dye tagged loci | | 2 | | No | ns | |  |
| Green color dye tagged loci vs Yellow color dye tagged loci | | -12 | | No | ns | |  |
| Green color dye tagged loci vs Red color dye tagged loci | | -2 | | No | ns | |  |
| Green color dye tagged loci vs Purple color dye tagged loci | | -2 | | No | ns | |  |
| Yellow color dye tagged loci vs Red color dye tagged loci | | 10 | | No | ns | |  |
| Yellow color dye tagged loci vs Purple color dye tagged loci | | 10 | | No | ns | |  |
| Red color dye tagged loci vs Purple color dye tagged loci | | 0 | | No | ns | |  |
| ns: non significant | | | | | | |  |

| Table S10: One way ANOVA (nonparameteric) for mean total peak heights of blue, green, yellow and red dye taged loci of POWERPLEX Y-23 SYSTEM MULTIPLEX KIT | | | |
| --- | --- | --- | --- |
| Friedman test |  |  |  |
| P value | 0.3916 |  |  |
| Exact or approximate P value? | Gaussian Approximation |  |  |
| P value summary | ns |  |  |
| Are means signif. different? (P < 0.05) | No |  |  |
| Number of groups | 4 |  |  |
| Friedman statistic | 3 |  |  |
|  |  |  |  |
| Dunn's Multiple Comparison Test | Difference in rank sum | Significant? P < 0.05? | Summary |
| Blue color dye tagged loci  vs Green color dye  tagged loci | -4 | No | ns |
| Blue color dye tagged loci  vs Yellow color dye tagged loci | 8 | No | ns |
| Blue color dye tagged loci  vs Red colordye tagged loci | -6 | No | ns |
| Green color dye  tagged loci vs Yellow color dye tagged loci | 12 | No | ns |
| Green color dye  tagged loci vs Red colordye tagged loci | -2 | No | ns |
| Yellow color dye tagged loci vs Red colordye tagged loci | -14 | No | ns |
| ns: non significant | | | |
|  |  |  |  |

| Table S11: One way ANOVA (nonparameteric) for mean total peak heights of blue, green, yellow and red dye taged loci of AMPFLSTR YFILER MULTIPLEX KIT | | | |
| --- | --- | --- | --- |
| Friedman test |  |  |  |
| P value | 0.094 |  |  |
| Exact or approximate P value? | Gaussian Approximation |  |  |
| P value summary | ns |  |  |
| Are means signif. different? (P < 0.05) | No |  |  |
| Number of groups | 4 |  |  |
| Friedman statistic | 6.391 |  |  |
|  |  |  |  |
| Dunn's Multiple Comparison Test | Difference in rank sum | Significant? P < 0.05? | Summary |
| Blue color dye  tagged loci  vs Green color dye  tagged loci | -11 | No | ns |
| Blue color dye  tagged loci  vs Yellow color tagged loci | 11 | No | ns |
| Blue color dye  tagged loci  vs Red color dye tagged loci | -2 | No | ns |
| Green color dye  tagged loci vs Yellow color tagged loci | 22 | No | ns |
| Green color dye  tagged loci vs Red color dye tagged loci | 9 | No | ns |
| Yellow color tagged loci vs Red color dye tagged loci | -13 | No | ns |
| ns: non significant | | | |

| Table S12: One way ANOVA (nonparameteric) for mean total peak heights of blue, green, yellow, Red and Purple dye taged loci of AMPFLSTR YFILER PLUS MULTIPLEX KIT | | | | | | | |
| --- | --- | --- | --- | --- | --- | --- | --- |
| Friedman test | |  | |  | |  | |
| P value | | 0.052 | |  | |  | |
| Exact or approximate P value? | | Gaussian Approximation | |  | |  | |
| P value summary | | ns | |  | |  | |
| Are means signif. different? (P < 0.05) | | No | |  | |  | |
| Number of groups | | 5 | |  | |  | |
| Friedman statistic | | 9.391 | |  | |  | |
|  | |  | |  | |  | |
| Dunn's Multiple Comparison Test | | Difference in rank sum | | Significant? P < 0.05? | | Summary | |
| Blue color dye tagged loci  vs Green color dye tagged loci | | 16 | | No | | ns | |
| Blue color dye tagged loci  vs Yellow color dye tagged loci | | 22 | | No | | ns | |
| Blue color dye tagged loci  vs Red color dye tagged loci | | 3 | | No | | ns | |
| Blue color dye tagged loci  vs Purple color dye tagged loci | | -6 | | No | | ns | |
| Green color dye tagged loci vs Yellow color dye tagged loci | | 6 | | No | | ns | |
| Green color dye tagged loci vs Red color dye tagged loci | | -13 | | No | | ns | |
| Green color dye tagged loci vs Purple color dye tagged loci | | -22 | | No | | ns | |
| Yellow color dye tagged loci vs Red color dye tagged loci | | -19 | | No | | ns | |
| Yellow color dye tagged loci vs Purple color dye tagged loci | | -28 | | No | | ns | |
| Red color dye tagged loci vs Purple color dye tagged loci | | -9 | | No | | ns | |
| ns: non significant | | | | | | | |
| Table S13: One way ANOVA (nonparameteric) for mean total peak heights of blue, green, yellow and red dye taged loci of INVESTIGATOR ARGUS X-12 MULTIPLEX KIT | | | | | | |  |
| Friedman test |  | |  | |  | |  |
| P value | 0.0752 | |  | |  | |  |
| Exact or approximate P value? | Gaussian Approximation | |  | |  | |  |
| P value summary | ns | |  | |  | |  |
| Are means signif. different? (P < 0.05) | No | |  | |  | |  |
| Number of groups | 4 | |  | |  | |  |
| Friedman statistic | 6.9 | |  | |  | |  |
|  |  | |  | |  | |  |
| Dunn's Multiple Comparison Test | Difference in rank sum | | Significant? P < 0.05? | | Summary | |  |
| Blue color dye tagged loci  vs Green color dye tagged loci | -10 | | No | | ns | |  |
| Blue color dye tagged loci  vs Yellow color dye tagged loci | -4 | | No | | ns | |  |
| Blue color dye tagged loci  vs Red color dye tagged loci | -22 | | No | | ns | |  |
| Green color dye tagged loci vs Yellow color dye tagged loci | 6 | | No | | ns | |  |
| Green color dye tagged loci vs Red color dye tagged loci | -12 | | No | | ns | |  |
| Yellow color dye tagged loci vs Red color dye tagged loci | -18 | | No | | ns | |  |
| ns: non significant | | | | | | |  |
